# Sex-Specific Thermal Adaptation in *Riptortus pedestris*: Integrating Logistic Thresholds and Transcriptomic Responses

**DOI:** 10.3390/biology15070552

**Published:** 2026-03-30

**Authors:** Liyan Zhang, Yuxin Zhou, Xuechao Zhou, Xiaofeng Li, Yulong Niu, Zhengxiao Du, Wu Zhang, Yu Gao

**Affiliations:** 1 College of Plant Protection, Jilin Agricultural University, Changchun 130118, China; 2 State Key Laboratory of Green Pesticide, Guizhou University, Guiyang 550025, China; 3 Chifeng Agricultural and Animal Husbandry Scientific Research Institute, Chifeng 024050, China; 4 Heihe Branch of Heilongjiang Academy of Agricultural Sciences, Heihe 164399, China; 5 Key Laboratory of Soybean Disease and Pest Control, Ministry of Agriculture and Rural Affairs, Changchun 130118, China

**Keywords:** *Riptortus pedestris*, thermal thresholds, heat shock protein, soybean pest

## Abstract

The bean bug *Riptortus pedestris* (Hemiptera: Alydidae) is a major soybean pest in Asia, increasingly threatened by rising temperatures. It is crucial to clarify the high-temperature lethal thresholds of *R. pedestris* for predicting population dynamics under climate warming. Previous physiological data showed that female adults have significantly higher heat tolerance than males, with HLT_50_ values of 39.76 °C versus 38.45 °C and HLT_90_ values of 42.99 °C versus 42.44 °C. This sex-specific difference suggests distinct molecular responses to thermal stress. To test this hypothesis, we performed transcriptome sequencing of adults exposed to 24 °C (control), 40 °C (sublethal), and 44 °C (lethal), with males and females treated separately. A high-quality assembly yielded 270,199 unigenes, with 71 heat shock protein (HSP) genes identified across six subfamilies (sHSP, HSP40, HSP60, HSP70, HSP90, HSFs). HSPs exhibited a dual strategy: constitutive expression for basal proteostasis and strong induction—especially at 44 °C—for stress defense. Notably, responses were sexually dimorphic: females upregulated multiple stress-responsive HSPs (e.g., *RpedHsp15.5-3*, *RpedHsp30.8*) to protect reproduction, while males specifically induced *RpedHsp83.6*, possibly for signaling regulation. Phylogenetic analysis confirmed conservation within Hemiptera. These findings reveal the molecular basis of thermal adaptation in *R. pedestris* and identify key HSPs as potential targets for RNAi, HSP inhibitors, or precision thermal control.

## Simple Summary

*Riptortus pedestris* (Hemiptera: Alydidae) is one of the most serious pests affecting soybean quality and yield. In this study, we defined thermal thresholds via survival modeling, then exposed adult bugs to normal (24 °C) and high temperatures (40 °C and 44 °C) and analyzed their gene expression using RNA sequencing. The results showed that high temperatures significantly reduced survival of *R. pedestris*. The heat shock protein (HSP) genes were strongly activated under extreme heat, with distinct patterns between males and females—females showed broader HSP upregulation, likely to protect reproduction, while males relied on specific HSPs like *RpedHsp83.6*, which may support male-specific physiological functions. These results reveal how *R. pedestris* copes with heat stress at the molecular level and highlight potential targets for eco-friendly pest control strategies in a warming climate.

## 1. Introduction

The bean bug, *Riptortus pedestris* (Hemiptera: Alydidae), is an important pest in legume fields throughout East and Southeast Asia (including China, Bangladesh, India, Japan, and Korea) [1,2,3,4,5]. It is the primary agent of soybean stay-green syndrome in North China [6,7,8]. *R. pedestris* feeds by piercing and sucking the sap from pods and seeds [4]. As global warming continues, its destructive activity is expected to intensify, which will erode both soybean yield and quality, resulting in severe economic losses [9,10]. Previous studies found that temperature exerts a significant influence on the growth, development and reproduction of *R. pedestris* under both constant and fluctuating temperature conditions, thereby indirectly influencing the population growth rate [9,11]. Moreover, temperatures affect the metabolism of *R. pedestris*. Specifically, high temperatures have been found to significantly impact its body weight, carbon dioxide emissions, nutrient substances, nutrient metabolic enzymes, and antioxidant enzymes [10]. While recent surveys have identified several emerging pests threatening soybean production in China [12], *R. pedestris* has re-emerged as a particularly severe and widespread threat, warranting urgent mechanistic investigation. Thus, temperature and its dynamic changes, along with their long-term effects on insect populations, must be accounted for in effective pest management. However, the molecular mechanisms underlying the response of *R. pedestris* to temperature stress are not yet understood.

Transcriptome sequencing analysis serves as an important tool for investigating the genetic basis and molecular mechanisms underlying temperature adaptation [13]. Transcriptional plasticity plays a key role in driving variations in temperature tolerance limits across different species. The responses of various insects to thermal stress not only exhibit a degree of conservatism but also demonstrate species-specific characteristics. For example, hydrogen sulfide exposure was shown to alter gene expression in *Bombyx mori*, enhancing energy metabolism, material transport, and silk protein synthesis while suppressing inflammation, thereby improving growth and cocoon-related economic traits; glyphosate exposure was found to disrupt gene transcription and immune function in *B. mori*; high temperature induced distinct whole-genome expression profiles in two color morphs of *Acyrthosiphon pisum*; and both heat and cold stress triggered transcriptomic responses in *Cryptolaemus montrouzieri* [14,15,16,17]. Insects respond to heat stress by upregulating the expression of multiple genes, including heat shock proteins, heat shock transcription factors, HSR-omega proteins, and phosphoglucose isomerase genes [18,19,20,21]. *R. pedestris* is highly sensitive to thermal variation, with temperature known to significantly influence its development, survival, metabolism, and population dynamics. Empirical studies have established clear thermal thresholds for its survival and reproduction [22,23], underscoring its suitability as a model for investigating molecular responses to heat stress. This study will employ transcriptomic sequencing to analyze the adaptive responses of *R. pedestris* to three temperature conditions. Heat shock proteins (HSPs) are a class of evolutionarily conserved molecular chaperones that are rapidly upregulated when organisms encounter adverse environmental stresses. Widely present across all domains of life, HSPs serve as key molecular markers of thermal stress in insects. They help maintain cellular proteostasis by assisting in the refolding of misfolded proteins or facilitating the degradation of irreversibly damaged proteins, thereby preventing toxic protein aggregation [24,25,26]. Through these mechanisms, HSPs play a critical role in mitigating cellular damage and enhancing organismal resilience under stress conditions. The findings would enhance our understanding of how *R. pedestris* adapts to temperature changes and promoting the pest management of leguminous crops. Therefore, this study aims to: (1) Determine sex-specific survival rates of *R. pedestris* adults under varying temperature–time regimes, establish a high-temperature zonation system, and define thermal tolerance thresholds, providing the physiological basis for subsequent molecular analyses; (2) Construct a transcriptome database at key thermal thresholds and identify differentially expressed genes to delineate the global transcriptional response to heat stress; (3) Focus specifically on the HSP gene family to characterize its composition, evolutionary features, and expression dynamics, providing a foundation for understanding its potential role and sex-biased regulation in thermal adaptation.

## 2. Materials and Methods

### 2.1. Survival Rate Determination at Different Temperatures

The *R. pedestris* population was collected from soybean fields in Changchun, Jilin Province, China, and reared in the laboratory for at least two consecutive generations on fresh soybean pods in the intelligent artificial climate chamber (PGX-500D, Zhongyi Guoke (Beijing) Technology Co., Ltd., Beijing, China) (24 ± 1 °C, photoperiod L:D = 16:8 h, relative humidity 60–70%).

In the field, high temperatures typically occur between 12:00 and 16:00, lasting approximately 4 h. Considering regional differences in China, high-temperature durations in major soybean-producing areas are as follows: 1–2 h in Northeast China, 2–3 h in the Huang-Huai-Hai and Northwest regions, and 3–4 h in Southern China. Accordingly, we designed short-term heat stress experiments to further investigate the survival of *R. pedestris* under temperatures ranging from 32 °C to 44 °C and exposure durations of 1–4 h. The control treatment was maintained at 24 ± 1 °C while experimental treatments included 32 ± 1 °C, 36 ± 1 °C, 40 ± 1 °C, 42 ± 1 °C, and 44 ± 1 °C, each applied for 1, 2, 3, or 4 h. These temperatures were selected based on the known thermal biology of *R. pedestris*: 32 °C and 36 °C represent moderate heat stress approaching the optimal range upper limit; 40 °C and 42 °C corresponds to the critical thermal maximum previously reported for this species; 44 °C represent severe heat stress. Each treatment group consisted of 30 adult insects, with three biological replicates [7,10]. After heat exposure, male and female adults were returned to ambient conditions for normal rearing. Survival was assessed at 1 h and 24 h post-treatment. An insect was considered dead if it showed no movement in the body, antennae, or legs upon gentle prodding with a dissecting needle. Mortality counts were recorded for each treatment group.

### 2.2. Insect Source and Tissue Sampling

Healthy adult insects aged three days post-eclosion were selected for this study. All insects were derived from a laboratory colony maintained under standardized conditions for multiple generations and fed fresh soybean pods. Only individuals that were actively feeding, showed normal mobility, and exhibited no visible morphological abnormalities or signs of poor health were used. The selected temperatures are based on the results in Section 3.1. Three temperature treatments were 24 °C (normal control), 40 °C (representing the sublethal high temperature), and 44 °C (representing the lethal high temperature). Both female and male adults were collected at each temperature, resulting in six experimental groups with three biological replicates per group (each replicate consisting of three adults): A1–A3 (24 °C females), B1–B3 (24 °C males), C1–C3 (40 °C females), D1–D3 (40 °C males), E1–E3 (44 °C females), and F1–F3 (44 °C males). All temperature treatments were conducted in environmental chambers (Model: GXZ/RXZ Series, Manufacturer: Ningbo Southeast Instrument Co., Ltd., Ningbo, China) with both temperature and relative humidity (RH) controlled at relative humidity 60–70%, which represents the average humidity conditions in local soybean fields during summer [7]. This humidity level was maintained constant across all temperature treatments to ensure that observed effects were attributable solely to thermal stress. All samples were exposed to the respective temperatures for 4 h, immediately flash-frozen in liquid nitrogen, and stored at –80 °C for subsequent RNA extraction.

### 2.3. Sample Preparation and High-Throughput Sequencing and Transcriptome Sequencing

Total RNA was extracted using TRIzol^®^ Reagent (Invitrogen, Carlsbad, CA, USA) following the manufacturer’s instructions. RNA concentration and purity were measured with a NanoDrop 2000 spectrophotometer (Thermo Fisher Scientific, Waltham, MA, USA), and RNA integrity was assessed by 1% agarose gel electrophoresis and an Agilent 2100 Bioanalyzer (Santa Clara, CA, USA), with only samples exhibiting an RNA Integrity Number (RIN) > 8.0 used for subsequent analysis. Qualified RNA samples were sent to Sangon Biotech Co., Ltd (Shanghai, China). for library preparation and paired-end sequencing (PE150) on an Illumina (San Diego, CA, USA) NovaSeq 6000 platform.

### 2.4. RNA Extraction and Transcriptome Processing and DEG Identification

To investigate the gene expression responses of *R. pedestris* under different temperature stresses, raw sequencing reads were first subjected to quality control using fastp (v0.23.2) to remove adapter sequences, poly-N tails, and low-quality bases (Phred score < 20); reads shorter than 50 bp after trimming were discarded. Clean reads were then mapped to the reference transcriptome assembly using Bowtie2 (v2.3.2), and gene expression levels were quantified as raw read counts. These counts were normalized to account for differences in library size and composition. A hierarchical clustering heatmap was generated based on log_10_-transformed gene expression counts obtained from the transcriptome sequencing data, using the default settings of the online platform (Biomarker Technologies Co., Ltd. Beijing, China).

Concurrently, to systematically identify key responsive genes in *R. pedestris* across different temperature and sex conditions, differential expression analysis was performed using the DESeq2 package (v1.12.4) in R (v3.3.1) based on the raw count data. Differentially expressed genes (DEGs) were defined using the criteria: q-value < 0.005 and |log_2_(Fold Change)| > 2. These thresholds were selected to balance statistical rigor and biological relevance: a stringent q-value (<0.005) effectively controls the false discovery rate in multiple testing, while a |log_2_FC| > 2 cutoff (corresponding to a ≥4-fold expression change) focuses on genes with substantial transcriptional responses likely to underlie critical thermal adaptation mechanisms—such as HSPs—and is commonly applied in insect transcriptomic studies. Pairwise comparisons were specifically conducted between all three temperature groups: 24 °C vs. 40 °C, 24 °C vs. 44 °C, and 40 °C vs. 44 °C.

### 2.5. Phylogenetic Analysis of HSPs

Prior to phylogenetic reconstruction, heat shock protein (HSP) genes in *R. pedestris* were systematically identified and classified into families (sHSP, HSP40, HSP60, HSP70, HSP90, and HSF) based on the presence of canonical functional domains using the SMART database (http://smart.embl.de/, accessed on 11 January 2026). The theoretical molecular weight (Mw) of each HSP was calculated from its amino acid sequence using the Protein Molecular Weight Calculator in the Sequence Manipulation Suite (SMS2; https://www.detaibio.com/sms2/protein_mw.html, accessed on 9 January 2026), and subcellular localization was predicted using DeepLoc-2.0 (https://services.healthtech.dtu.dk/service.php?DeepLoc-2.0,accessed on 26 December 2025). The identified heat shock protein (HSP) sequences from *R. pedestris* were aligned with homologous HSP sequences from well-annotated model insects—including *Drosophila melanogaster*, *Tribolium castaneum*, and *Nilaparvata lugens*—using MAFFT v7 with the L-INS-i algorithm. A maximum likelihood (ML) phylogenetic tree was constructed using IQ-TREE v2.2.0, with the best-fit amino acid substitution model automatically selected by built-in ModelFinder. Branch support was assessed using 5000 replicates of the ultrafast bootstrap method, and the resulting phylogenetic tree was visualized with FigTree v1.4.4 [27,28].

### 2.6. q-PCR Analysis

Total RNA was reverse-transcribed using the HiScript^®^ II Q RT SuperMix for qPCR (+gDNA wiper) kit (Vazyme Biotech Co., Ltd., Nanjing, China). The resulting cDNA was diluted 50-fold with RNase-free H_2_O and used as template for qRT-PCR to assess gene expression levels. Gene-specific primers are listed in Table S1. A 20 µL reaction volume was prepared according to the manufacturer’s instructions. qRT-PCR was performed on a CFX96™ Real-Time PCR Detection System (Bio-Rad Laboratories, Inc., California, USA) under the following cycling conditions: an initial denaturation at 95 °C for 3 min, followed by 40 cycles of 95 °C for 5 s and 60 °C for 30 s. The RPL7A gene of *R. pedestris* was used as an internal reference for normalization, and relative gene expression levels were calculated using the 2^−ΔΔCt^ method. Based on the study by Wang et al. [29], which identified RPS23 and RPL7A as the most stable reference genes in *R. pedestris* under temperature stress conditions, RPL7A was selected as the reference gene for qRT-PCR normalization. Primer pairs (listed in Table S1) were validated with amplification efficiencies of 90–110% and R^2^ > 0.99.

### 2.7. Data Analysis

Data were analyzed using SPSS Statistics 26 (IBM Inc., Chicago, IL, USA). One-way ANOVA was used to evaluate significant differences (*p* < 0.05) in adult survival rates among different temperature and duration treatments. Duncan’s new multiple range test was applied for post hoc comparisons. Mann–Whitney U test was conducted to assess sex-specific differences in survival under identical treatment conditions (*p* < 0.05). Logistic regression models were fitted to calculate the high lethal temperature for 50% mortality (HLT_50_) and 90% mortality (HLT_90_) for both sexes. Temperature zones were defined as follows: Lethal high-temperature zone: survival rate < 5%; Sublethal high-temperature zone: 5% ≤ survival rate < 95%; Upper limit of optimal temperature zone: survival rate ≥ 95%.

## 3. Results

### 3.1. Survival Rates and Classification of High-Temperature Zones in R. pedestris

**Figure 1:**
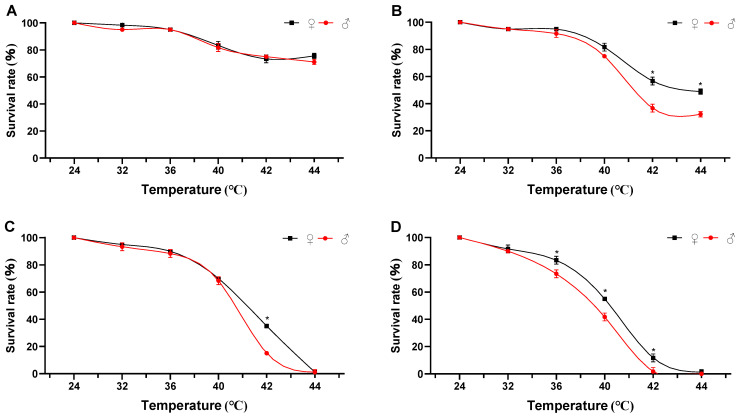
Survival rates of *R. pedestris* under different temperature and time treatments (recovery for 24 h). (**A**) Survival rate after 1 h of heat exposure (24–44 °C). (**B**) Survival rate after 2 h of heat exposure (24–44 °C). (**C**) Survival rate after 3 h of heat exposure (24–44 °C). (**D**) Survival rate after 4 h of heat exposure (24–44 °C). Note: The asterisk indicates the significant difference between males and females.

When exposure duration was held constant, survival rates after 24 h of recovery at room temperature decreased significantly with increasing temperature (Figure 1). Significant differences in survival were observed across temperatures for all exposure durations (1–4 h). Specifically: At 1 h, 2 h, and 3 h exposures, survival dropped significantly when temperature reached 36 °C or higher (1 h: ♀: *F* = 86.606, *p* < 0.001, *df* = 5; ♂: *F* = 215.015, *p* < 0.001, *df* = 5; 2 h: ♀: *F* = 417.755, *p* < 0.001, *df* = 5; ♂: *F* = 802.191, *p* < 0.001, *df* = 5; 3 h: ♀: *F* = 7539.40, *p* < 0.001, *df* = 5; ♂: *F* = 1137.555, *p* < 0.001, *df* = 5). At 4 h exposure, survival declined significantly starting at 32 °C (♀: *F* = 1119.594, *p* < 0.001, *df* = 5; ♂: *F* = 1374.133, *p* < 0.001, *df* = 5). In addition, significant differences in survival rates between males and females were found under thermal stress conditions of 36 °C (4 h), 40 °C (4 h), 42 °C (2, 3, 4 h), and 44 °C (2 h) (Table S2). Female adults exhibited higher thermal tolerance than males, with HLT_50_ and HLT_90_ values 1.31 °C and 0.55 °C higher, respectively. Our results demonstrated that the sensitivity of *R. pedestris* to heat stress increased significantly with prolonged exposure duration, exhibiting a clear time-dependent enhancement in heat sensitivity. Under exposure durations of 1, 2, and 3 h, the survival rate of *R. pedestris* decreased significantly when the temperature rose to 36 °C and above, with differences observed between male and female adults at this temperature threshold (95% confidence intervals for all data points are provided in Supplementary Table S3). However, when the exposure duration was extended to 4 h, the critical temperature inducing a significant decline in survival rate dropped to 32 °C. This indicates a significant synergistic lethal effect between high-temperature stress intensity and exposure duration in *R. pedestris*, and that prolonged exposure substantially lowers the critical temperature threshold for heat tolerance in this pest.

Mortality increased with both temperature and exposure duration. After 4 h of heat stress followed by 24 h of recovery at room temperature, mortality data for both sexes were fitted to logistic curves (Table 1). The adjusted R^2^ values of all fitted models exceeded 0.98, indicating excellent model fit for describing temperature-dependent mortality dynamics.

Based on the fitted survival curves, high-temperature zones for adult *R. pedestris* were delineated as follows: The upper limit of the optimal temperature zone was 34.73 °C for females and 31.68 °C for males. The sublethal high-temperature zone ranged from 34.73 °C to 43.77 °C in females and from 32.14 °C to 43.24 °C in males. The lower threshold of the lethal high-temperature zone was 43.77 °C for females and 43.24 °C for males (Table 1). HLT_50_ and HLT_90_ values were derived from the logistic models: females: HLT_50_ = 39.76 °C, HLT_90_ = 42.99 °C; males: HLT_50_ = 38.45 °C, HLT_90_ = 42.44 °C. Notably, female adults exhibited higher thermal tolerance than males, with HLT_50_ and HLT_90_ values 1.31 °C and 0.55 °C higher, respectively.

### 3.2. Transcriptome Sequencing and Assembly Quality Assessment

In this study, transcriptome sequencing was performed on six samples of *R. pedestris* adults—three females and three males—collected at 24 °C, 40 °C, and 44 °C (labeled A–F). Using the Illumina NovaSeq 6000 platform, we obtained 47,437,106 to 59,921,308 raw reads per sample, corresponding to 7.12–8.99 Gb of data. The GC content ranged from 45.28% to 47.12%, and all Q30 values exceeded 88.85% (with a maximum of 91.67%) (Table S4), indicating high sequencing quality suitable for downstream analyses.

All clean reads were pooled for *de novo* transcriptome assembly, yielding a total of 443,995 transcripts and 270,199 non-redundant unigenes, with total lengths of 244,280,162 bp and 131,166,405 bp, respectively. The average lengths were 550.19 bp for transcripts and 485.44 bp for unigenes, with N50 values of 720 bp and 575 bp, respectively (Table S5). Among these, 23,475 unigenes (8.69% of the total) were ≥1000 bp in length.

BUSCO assessment confirmed high assembly completeness: 93.0% of conserved insect orthologs were recovered (77.0% single-copy and 16.0% duplicated), with only 4.5% fragmented and 2.5% missing (out of *n* = 1367 BUSCO genes), further validating the high quality and completeness of the assembly.

For comprehensive functional annotation, unigenes were aligned against multiple public databases. The highest number of annotations was achieved in the NR database (81,356 unigenes), followed by NT (99,470), Swiss-Prot (71,269), and KOG (41,804). Notably, longer sequences (≥1000 bp) showed significantly higher annotation efficiency than shorter ones (<300 bp). For instance, in Swiss-Prot and NR, 14,415 and 16,297 unigenes ≥ 1000 bp were annotated, respectively, whereas although there were 24,910 and 27,129 unigenes < 300 bp, their annotation rates were markedly lower. This highlights the critical influence of sequence length and assembly integrity on functional annotation success (Table S6). Together, key quality indicators including high Q30 (>88.85%), unigene N50 of 575 bp, and 93.0% BUSCO completeness are consistent with or exceed those reported in recent transcriptomic studies of Hemipteran insects, confirming that our assembly meets the standard required for reliable gene annotation and expression analysis [30,31].

Collectively, this study has established a high-quality, dual-factor (temperature × sex) reference transcriptome database for *R. pedestris*, providing a robust foundation for in-depth investigation of its molecular responses to heat stress and sex-specific expression patterns.

### 3.3. Identification of Differentially Expressed Genes in R. pedestris Under Temperature Stress

**Figure 2:**
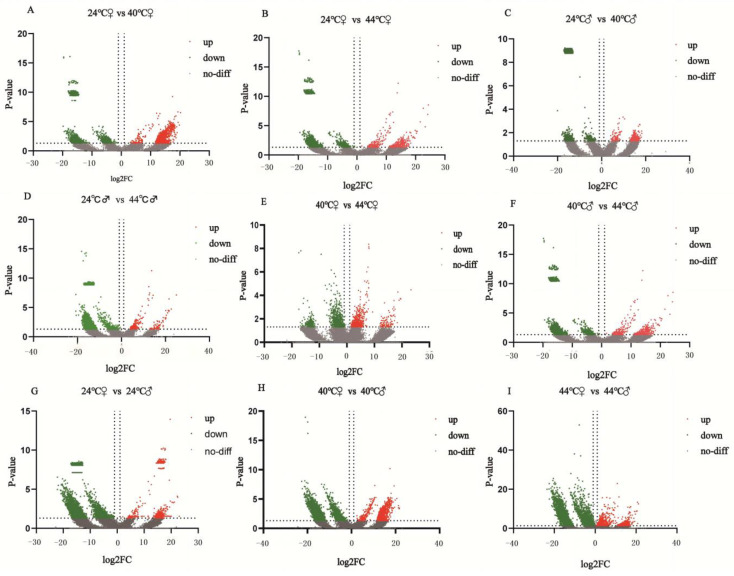
This figure presents volcano plots of the global gene expression in *R. pedestris* adults under treatments at 24 °C, 40 °C, and 44 °C, visualizing the differential expression characteristics among different temperature conditions. Panels (**A**–**I**) correspond to comparisons between different temperature groups: Comparisons between different temperatures at the same sex (**A**–**D**): including 24 °C vs. 40 °C and 24 °C vs. 44 °C; Comparisons between 40 °C and 44 °C at the same sex (**E**,**F**); Comparisons between different sexes at the same temperature (**G**–**I**). In these plots, the x-axis (log_2_FC) represents the fold change in expression levels between two groups; the further the value is from 0, the greater the difference in expression. The y-axis (*p*-value) indicates the statistical significance of the differences, with higher values representing greater confidence in the differential expression. Data points are color-coded as follows: red for sequences significantly upregulated (up), green for those significantly downregulated (down), and grey for those showing no significant difference (no-diff). The dashed lines indicate the thresholds for differential expression: the vertical dashed line represents a |log_2_FC| > 2 cutoff (corresponding to a ≥4-fold change), and the horizontal dashed line represents a significance cutoff of q-value < 0.005.

From each comparison, the top 10 most significantly upregulated and downregulated genes (i.e., those with the largest |log_2_FC| values) were extracted, resulting in a total of 180 ‘top’ differentially expressed genes (DEGs). The coding sequences of these genes were manually annotated via BLAST (NCBI, https://www.ncbi.nlm.nih.gov/orffinder/, accessed on 13 February 2026) searches against the NCBI database, and the results are summarized in Table S7 and Figure 2. All HSP sequences are listed in Data S1.

Among these 180 top DEGs, approximately 63% (114 genes) were annotated as ‘hypothetical protein’ or ‘uncharacterized protein.’ Although these poorly characterized genes may include species-specific stress-responsive factors, their ambiguous functional annotations limit direct mechanistic interpretation and necessitate further experimental validation or homology-based modeling for functional elucidation.

While some annotated DEGs—such as cytoskeletal proteins (e.g., α/β-tubulin), metabolic enzymes (e.g., cytosol aminopeptidase), immune-related molecules (e.g., thanatin), and transporters (e.g., Tret1)—show altered expression under heat stress [13,32,33,34,35,36,37], most lack direct experimental evidence in insect thermal responses, and their expression patterns show limited consistency across sexes or temperature gradients.

In contrast, comparisons between lethal high temperature (44 °C) and control (24 °C) in both females and males (A vs. E and B vs. F) revealed that the most strongly and consistently upregulated genes belong to the heat shock protein (HSP) family—including 70 kDa heat shock protein, heat shock cognate 71 kDa protein, heat shock protein 68-like, and molecular chaperone DnaK. These HSPs play a highly conserved and central role in maintaining proteostasis under heat stress and have been extensively validated as key regulators of thermotolerance in insects [38,39,40]. Given their robust induction, cross-sex consistency, and well-established biological function, we focused our subsequent analyses on the HSP gene family.

These findings highlight diverse molecular strategies employed by *R. Pedestris* to cope with elevated temperatures, with HSPs representing the most reliable and directly relevant markers of acute heat shock response.

### 3.4. Screening and Identification of HSP Genes in R. pedestris

Phylogenetic analysis of insect heat shock protein (HSP) families using the maximum likelihood method (model: PMB + G4; ultrafast bootstrap [UFBoot] = 5000 replicates) revealed that all HSP subfamily members in *R. pedestris* are highly conserved with those from Hemiptera and other closely related insect species.

Prior to phylogenetic reconstruction, HSP genes were identified and classified into families (sHSP, Hsp40, Hsp60, Hsp70, Hsp90) based on the presence of canonical functional domains using the SMART database (http://smart.embl.de/, accessed on 12 December 2025) [41].

**Figure 3:**
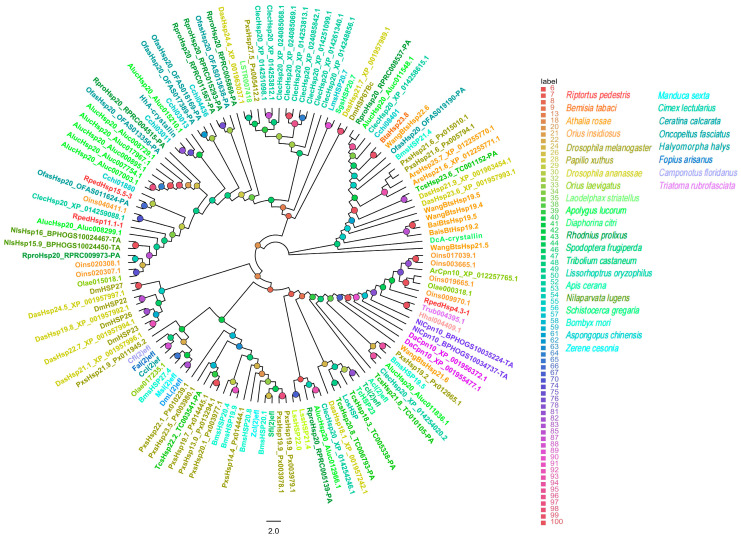
Homology analysis of sHSPs from *R. pedestris* and other representative insect species. The sequences used to construct the phylogenetic tree were derived from the following 29 species: *Bemisia tabaci* (Bta) [42,43,44]; *T. castaneum* (Tc) [41,42]; *H. halys* (Hh) [41,45]; *Athalia rosae* (Ar); *Drosophila ananassae* (Da); *N. lugens* (Nl) [42]; *B. mori* (Bm); *D. melanogaster* (Dm); *Papilio xuthus* (Px); *Laodelphax striatellus* (Ls); *Diaphorina citri* (Dc); *Schistocerca gregaria* (Sg); *Spodoptera frugiperda* (Sf); *Apis cerana* (Ac); *Lissorhoptrus oryzophilus* (Lo); *Zerene cesonia* (Zc); *Manduca sexta* (Ms); *Ceratina calcarata* (Cc); *Fopius arisanus* (Fa); *Camponotus floridanus* (Cf) [41]; *O. fasciatus* (Ofas); *R. prolixus* (Rpro); *Cimex lectularius* (Clec); *A. lucorum* (Aluc) [46]; *Aspongopus chinensis* (Cchi) [47]; *Triatoma rubrofasciata* (Trub); *Orius insidiosus* (Oins); *Orius laevigatus* (Olae) [45]. Bootstrap support values (5,000 replicates) are indicated by colored circles at the nodes (see legend).

Within the small HSP (sHSP) family (130 sequences), three *R. pedestris* members were distributed across distinct evolutionary clades; notably, *RpedHsp11.1-1* clustered with strong support alongside sHSPs from *Apolygus lucorum* and *N. lugens* (Figure 3).

**Figure 4:**
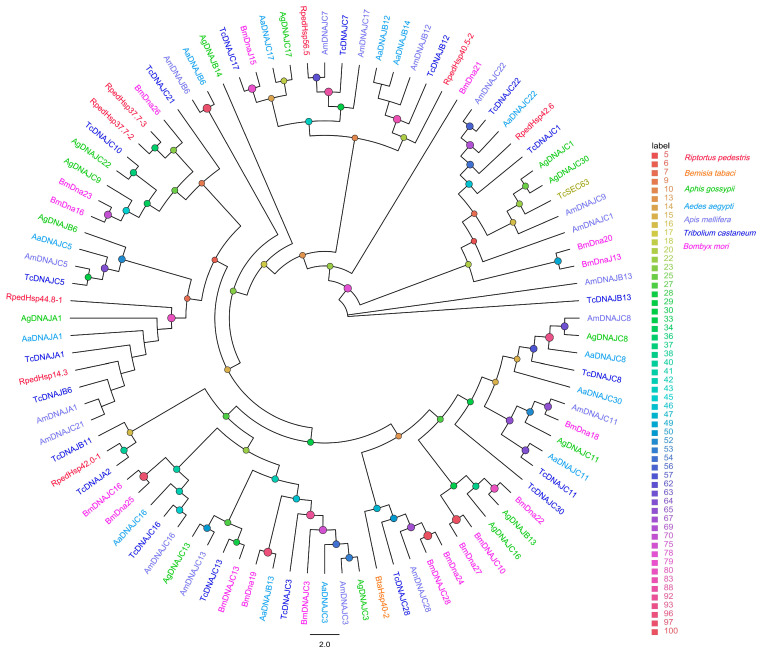
Homology analysis of HSP40s from *R. pedestris* and other representative insect species. The sequences used to construct the phylogenetic tree were derived from the following seven species: *B. tabaci* (Bta) [42]; *Aphis gossypii* (Ag); *Aedes aegypti* (Aa); *Apis mellifera* (Am); *T. castaneum* (Tc); *B. mori* (Bm) [41]. Bootstrap support values (5,000 replicates) are indicated by colored circles at the nodes (see legend).

In the HSP40/DnaJ family (92 sequences), seven members were clearly assigned to different DnaJ subtypes (Figure 4).

**Figure 5:**
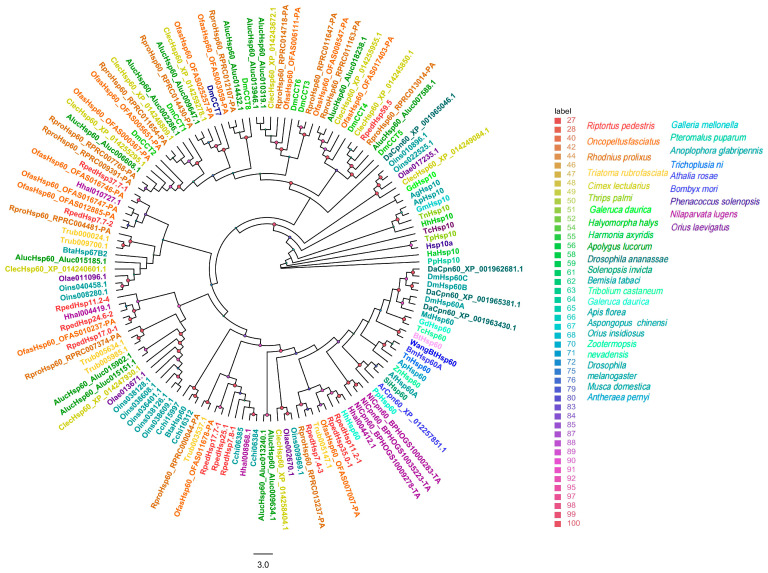
Homology analysis of HSP60s from *R. pedestris* and other representative insect species. The sequences used to construct the phylogenetic tree were derived from the following 34 species: *B. tabaci* (Bta) [42,43,44]; *T. castaneum* (Tc) [41,42]; *H. halys* (Hh) [41,45]; *A. rosae* (Ar); *D. ananassae* (Da); *N. lugens* (Nl) [42]; *B. mori* (Bm); *D. melanogaster* (Dm); *P. xuthus* (Px); *L. striatellus* (Ls); *D. citri* (Dc); *S. gregaria* (Sg); *S. frugiperda* (Sf); *A. cerana* (Ac); *L. oryzophilus* (Lo); *Z. cesonia* (Zc); *M. sexta* (Ms); *C. calcarata* (Cc); *F. arisanus* (Fa); *C. floridanus* (Cf) [41]; *O. fasciatus* (Ofas); *R. prolixus* (Rpro); *C. lectularius* (Clec); *A. lucorum* (Aluc) [46]; *A. chinensis* (Cchi) [47]; *T. rubrofasciata* (Trub); *O. insidiosus* (Oins); *O. laevigatus* (Olae) [45]. Bootstrap support values (5,000 replicates) are indicated by colored circles at the nodes (see legend).

All ten identified genes in the HSP60 family (134 sequences) formed multiple well-supported clades (UFBoot = 72–100%) exclusively with hemipteran species such as *Rhodnius prolixus* and *Oncopeltus fasciatus* (Figure 5).

**Figure 6:**
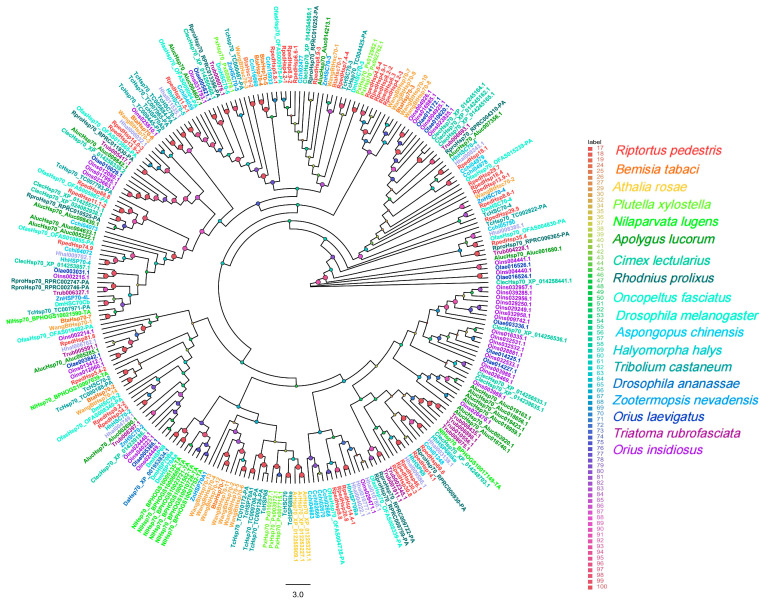
Homology analysis of HSP70s from *R. pedestris* and other representative insect species. The sequences used to construct the phylogenetic tree were derived from the following 18 species: *B. tabaci* (Bta) [42,43]; *T. castaneum* (Tc); *Plutella xylostella* (Px); *A. rosae* (Ar); *D. ananassae* (Da); *N. lugens* (Nl) [42]; *H. halys* (Hh); *Zootermopsis nevadensis* (Zn); *D. melanogaster* (Dm) [41]; *O. fasciatus* (Ofas); *R. prolixus* (Rpro); *C. lectularius* (Clec); *A. lucorum* (Aluc) [46]; *A. chinensis* (Cchi) [47]; *T. rubrofasciata* (Trub); *O. insidiosus* (Oins); *O. laevigatus* (Olae) [45]. Bootstrap support values (5,000 replicates) are indicated by colored circles at the nodes (see legend).

The HSP70 family (128 sequences) comprised 35 *R. pedestris* members, which were broadly dispersed across multiple functionally conserved branches (Figure 6).

**Figure 7:**
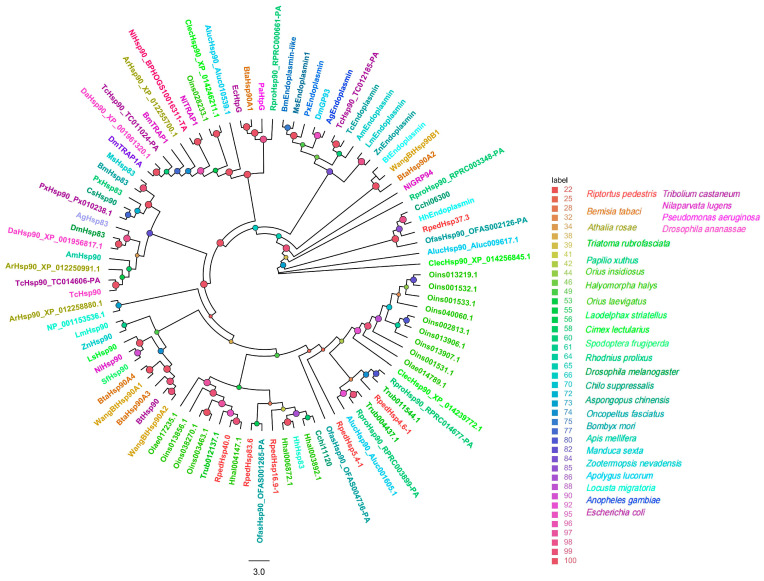
Homology analysis of HSP90s from *R. pedestris* and other representative insect species. The sequences used to construct the phylogenetic tree were derived from the following 29 species: *B. tabaci* (Bta) [41,42,43]; *T. castaneum* (Tc) [41,42]; *H. halys* (Hh) [41,45]; *A. rosae* (Ar); *D. ananassae* (Da); *N. lugens* (Nl) [42]; *D. melanogaster* (Dm); *P. xuthus* (Px); *L. striatellus* (Ls); *S. frugiperda* (Sf); *M. sexta* (Ms); *A. mellifera* (Am); *Locusta migratoria* (Lm); *Z. nevadensis* (Zn); *B. mori* (Bm); *Anopheles gambiae* (Ag); *Escherichia coli* (Ec); *Chilo suppressalis* (Cs) [41]; *O. fasciatus* (Ofas); *R. prolixus* (Rpro); *C. lectularius* (Clec); *A. lucorum* (Aluc) [46]; *A. chinensis* (Cchi) [47]; *T. rubrofasciata* (Trub); *O. insidiosus* (Oins); *O. laevigatus* (Olae) [45]. Bootstrap support values (5,000 replicates) are indicated by colored circles at the nodes (see legend).

In the HSP90 family (91 sequences), six members segregated into distinct conserved lineages: *RpedHsp83.6* grouped with its ortholog from *O. fasciatus*, while *RpedHsp16.9-1*, *RpedHsp40.0*, and *RpedHsp37.3* each formed highly supported clades (UFBoot ≥ 86%) with corresponding HSP90s from *Halyomorpha halys* (Figure 7).

Collectively, these results indicate that the HSP gene family in *R. pedestris* is primarily shaped by vertical inheritance, with each subfamily exhibiting strong structural and functional conservation. Moreover, the clear phylogenetic relationships reflect the evolutionary position of *R. pedestris* within the order Hemiptera.

### 3.5. Expression Patterns of HSP Genes in R. pedestris

According to Table S8, this study identified a total of 71 members of the heat shock protein (HSP) superfamily in *R. pedestris*, encompassing multiple classes including sHSP, HSP40, HSP60, HSP70, HSP90, and HSFs. The sHSP family comprises three members: *RpedHsp15.5-3* (15.5 kDa), *RpedHsp11.1-1* (11.1 kDa), and *RpedHsp4.3-1* (4.3 kDa), with predicted subcellular localizations in the cytoplasm, mitochondria, and extracellular space. The HSP40 family includes nine genes with molecular weights ranging from 14.3 to 56.5 kDa, all localized to the cytoplasm or co-localized with the periplasmic space. The HSP60 family consists of ten members, with molecular weights between 7.4 and 59.5 kDa, most of which are predicted to reside in the cytoplasm, while some are also associated with the periplasmic space. The HSP70 family is the largest group, containing 42 genes with an exceptionally broad molecular weight range (4.2–88.8 kDa). It includes high-molecular-weight canonical HSP70s (e.g., *RpedHsp74.9*, *RpedHsp88.8*, *RpedHsp81.8*) as well as numerous small or truncated isoforms (e.g., *RpedHsp30.8*, *RpedHsp20.8*, *RpedHsp18.1/18.4*, *RpedHsp14.4-1*), the majority of which are cytoplasmic, though a few are annotated as localized to the outer membrane, inner membrane, or periplasmic space. The HSP90 family comprises six members with molecular weights ranging from 4.6 to 83.6 kDa, including the full-length *RpedHsp83.6* (83.6 kDa) and several smaller variants (*RpedHsp16.9-1*, *RpedHsp5.4-1*, *RpedHsp4.6-1*, etc.), all primarily cytoplasmic. Additionally, two heat shock factor (HSF) genes—*RpedHsp71.9* and *RpedHsp25.3*—were identified, localized to the outer membrane and cytoplasm/inner membrane, respectively.

**Figure 8:**
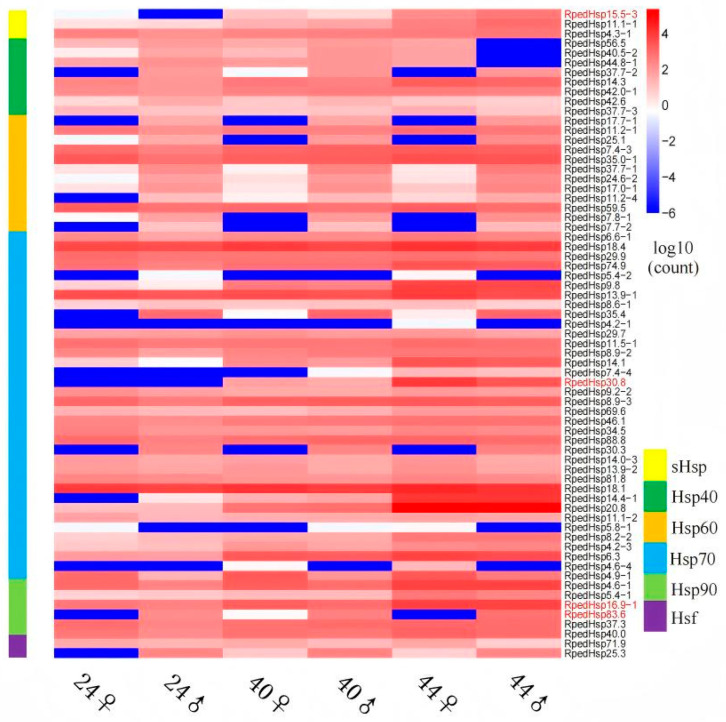
Heatmap showing the expression profiles of heat shock protein (HSP) genes in *R. pedestris* under three temperature treatments (24 °C, 40 °C, and 44 °C) across both sexes (♂ and ♀). The color scale ranges from blue (low expression) to red (high expression), representing log_10_(count)-normalized expression values, which visually illustrate the variation in gene expression levels across different experimental conditions. Horizontal axis: Experimental treatment groups, combining three temperature levels (24 °C, 40 °C, 44 °C) and two sexes (♂, ♀). Vertical axis: Differentially expressed HSP genes. Color bar on the left: Indicates the HSP subfamily to which each gene belongs—sHsp, Hsp40, Hsp60, Hsp70, or Hsp90.

**Figure 9:**
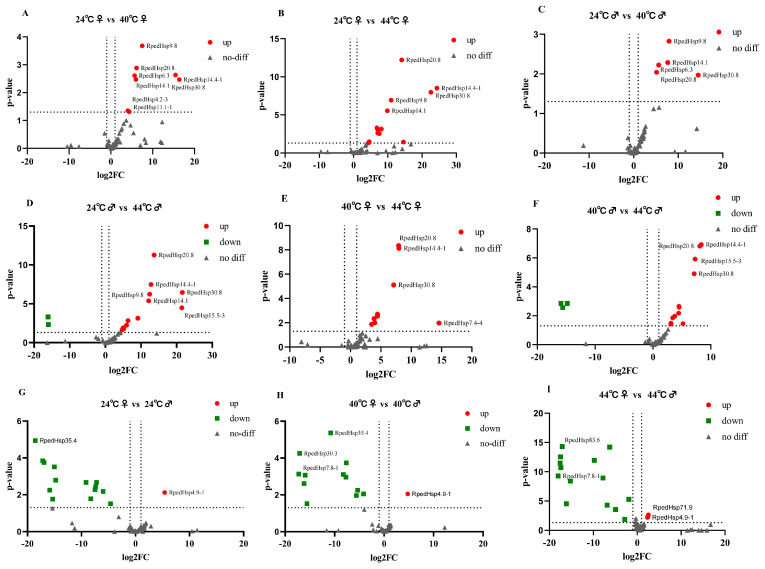
This figure presents volcano plots of heat shock protein (HSP) gene expression in *R. pedestris* under treatments at 24 °C, 40 °C, and 44 °C, visualizing the differential expression characteristics among different temperature conditions. Panels (**A**–**I**) correspond to comparisons between different temperature groups: Comparisons between different temperatures within the same sex (**A**–**D**): including 24 °C vs. 40 °C and 24 °C vs. 44 °C; Comparisons between 40 °C and 44 °C within the same sex (**E**,**F**). Comparisons between different sexes at the same temperature (**G**–**I**). In these plots: The x-axis (log_2_FC) represents the fold change in expression levels between two groups; the further the value is from 0, the greater the difference in expression. The y-axis (*p*-value) indicates the statistical significance of the differences, with higher values representing greater confidence in the differential expression. Data points are color-coded as follows: red for sequences significantly upregulated (up), green for those significantly downregulated (down), and grey for those showing no significant difference (no-diff). The dashed lines indicate the thresholds for differential expression: the vertical dashed line represents a |log_2_FC| > 2 cutoff (corresponding to a ≥4-fold change), and the horizontal dashed line represents a significance cutoff of q-value < 0.005.

**Figure 10:**
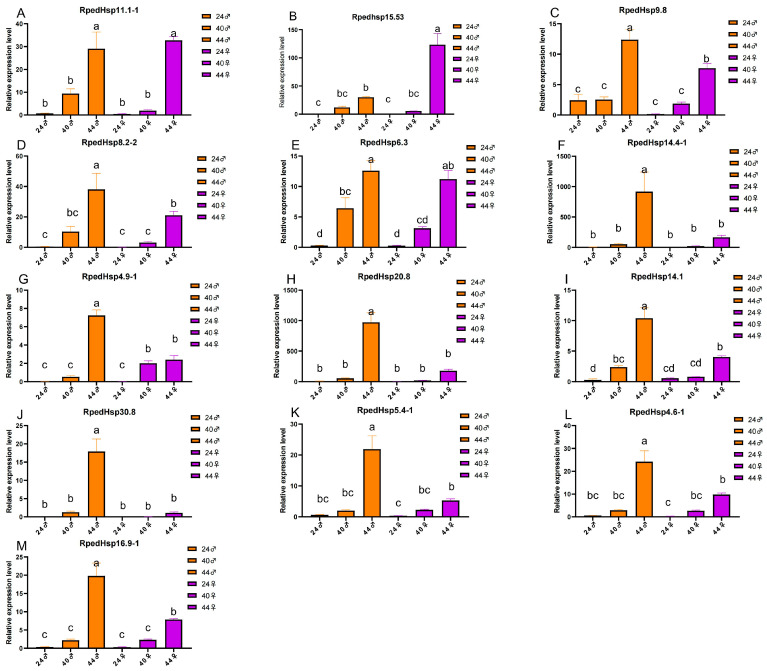
**Relative expression levels of 13 upregulated RpedHsp genes under heat stress, analyzed by quantitative real-time PCR (qRT-PCR).Panels A–M correspond to individual** *R. Pedestris* **HSPs genes**. (**A**) RpedHsp11.1-1; (**B**) RpedHsp15.53; (**C**) RpedHsp9.8; (**D**) RpedHsp8.2-2; (**E**) RpedHsp4.6-1; (**F**) RpedHsp16.9-1; (**G**) RpedHsp20.8; (**H**) RpedHsp14.1; (**I**) RpedHsp30.8; (**J**) RpedHsp5.4-1; (**K**) RpedHsp4.9-1; (**L**) RpedHsp6.3; (**M**) RpedHsp14.4-1. Relative expression levels of upregulated RpedHsp genes (e.g., *RpedHsp9.8*, *RpedHsp82.2*, *RpedHsp11.1*) under three temperature conditions (24 °C, 40 °C, and 44 °C) across different treatment durations and sexes. Columns represent distinct treatment combinations as indicated in the legend. Values are means ± standard error (SE) of *n* = 3 biological replicates. Statistical analysis was performed using one-way analysis of variance (ANOVA) followed by post hoc tests. Different lowercase letters (a–d) above bars denote significant differences among groups (*p* < 0.05); groups sharing the same letter are not significantly different. Most RpedHsp genes show markedly higher expression at 40 °C or 44 °C compared to 24 °C, with notable variation across temperature, time, and sex.

Based on expression profiles across the six temperature–sex conditions (Groups A–F: 24 °C female/male, 40 °C female/male, 44 °C female/male), the HSP superfamily in *R. pedestris* displayed highly divergent expression patterns in response to different temperature regimes (Figure 8, Figure 9 and Figure 10).

Within the sHSP family, *RpedHsp4.3-1* showed relatively high basal expression at 24 °C and was further upregulated with increasing temperature, whereas *RpedHsp11.1-1* and *RpedHsp15.5-3* were markedly induced only at 44 °C—particularly the latter, which surged more than 560-fold in males. qPCR validation confirmed that both *RpedHsp11.1-1* and *RpedHsp15.5-3* are strongly induced by heat stress at 44 °C, with pronounced upregulation primarily observed in females (Figure 10A,B).

The HSP60 family displayed subtype-specific responses: *RpedHsp42.0-1* was progressively upregulated with rising temperature; *RpedHsp14.3* was strongly activated specifically in females at 44 °C; in contrast, *RpedHsp44.8-1* was completely silenced in males at 44 °C, suggesting potential functional compensation by other paralogs. Meanwhile, *RpedHsp37.7-2/3* peaked in males at 40 °C, indicating heightened sensitivity to moderate heat stress.

Most members of the HSP60 family were constitutively and highly expressed under normal conditions (e.g., *RpedHsp59.5*, *RpedHsp35.0-1*), with only a few small-subunit genes—such as *RpedHsp25.1*—significantly upregulated in males at 44 °C.

The HSP70 family exhibited the most dramatic and diverse responses. Canonical HSC70 (*RpedHsp74.9*) and HSP110-type members (*RpedHsp88.8*, *RpedHsp81.8*) showed sustained upregulation under heat stress. In contrast, several truncated isoforms displayed extreme induction: *RpedHsp20.8* was highly expressed in females at 44 °C; *RpedHsp30.8* was almost exclusively expressed at 44 °C; and *RpedHsp18.1/18.4*, *RpedHsp14.4-1*, and *RpedHsp9.8* were all substantially upregulated in females at 44 °C—collectively highlighting a stronger heat-defense capacity in females. A few members (e.g., *RpedHsp5.4-2*) remained consistently lowly expressed across all conditions. qPCR analysis further validated the strong thermal induction of multiple HSP70 genes-including *RpedHsp9.8*, *RpedHsp8.2-2*, *RpedHsp6.3*, *RpedHsp14.4-1*, *RpedHsp4.9-1*, *RpedHsp20.8*, *RpedHsp14.1* and *RpedHsp30.8*—at 40 °C or 44 °C, with particularly robust responses detected in males (Figure 10C–J).

In the HSP90 family, the full-length isoform *RpedHsp83.6* was highly expressed only in males at 44 °C (log_2_FC ≈ 634), revealing pronounced sexual dimorphism. Conversely, short-form members—including *RpedHsp16.9-1*, *RpedHsp4.6-1*, and *RpedHsp5.4-1*—were strongly induced at 44 °C, predominantly in females. qPCR results corroborated the significant upregulation of *RpedHsp5.4-1*, *RpedHsp4.6-1*, and *RpedHsp16.9-1* under high-temperature stress, with dominant expression observed in males (Figure 10K,L,M).

Overall, *R. pedestris* employs a coordinated yet functionally partitioned HSP network to maintain basal proteostasis while simultaneously establishing a multi-layered, sex-dimorphic defense system against thermal stress. The consistency between qPCR and transcriptomic data for key thermoresponsive genes underscores the robustness of our findings.

## 4. Discussion

Under the backdrop of global warming, summer daily maximum temperatures in China’s major soybean-producing regions frequently reach 32–40 °C, with extremes exceeding 42 °C [48]. The observed sex-specific differences in thermal tolerance—where females exhibit higher lethal thresholds than males—not only reflect distinct physiological strategies but also likely shape population-level responses to heat stress. This sets the stage for examining how molecular mechanisms, particularly the HSP system, underpin these divergent phenotypes [49,50]. Logistic modeling precisely delineated the functional partitioning of critical thermal limits, revealing significant sex-specific differences in heat tolerance (female HLT_50_ exceeded male by 1.31 °C). Specifically, the upper limits of the optimal temperature zone (34.73 °C for females and 31.68 °C for males) and the HLT_50_ values (39.76 °C and 38.45 °C, respectively) closely aligned with our transcriptomic sampling temperatures of 24 °C (control) and 40 °C (sublethal heat), whereas the lethal thresholds (43.77 °C and 43.24 °C) corresponded well to the 44 °C lethal treatment.

Heat shock proteins are a highly conserved class of molecular chaperones that assist cells in coping with various stress conditions, maintain proteostasis, and participate in numerous biological processes [51]. Based on molecular weight and functional characteristics, the HSP family is primarily classified into sHSP, HSP40, HSP60, HSP70, HSP90, and HSP100 [52,53,54,55]. In this study, we performed transcriptome sequencing and systematic analyses of female and male *R. pedestris* adults exposed to 24 °C (control), 40 °C (sublethal heat), and 44 °C (lethal heat). We comprehensively identified HSP family members and revealed their dynamic expression patterns and sex-specific responses under thermal stress. Our results demonstrate that different HSP subfamilies exhibit high functional diversification and coordinated regulation, collectively forming a multi-layered heat stress defense network. Compared with previous studies on other Heteroptera and soybean pests, this study presents a more systematic and comprehensive analysis of the expression patterns of the HSP family. Moreover, by integrating multiple temperature gradients and explicitly incorporating sex as a biological variable-with independent thermal treatments applied to males and females-it provides deeper insight into sex-specific HSP expression profiles and their potential functional divergence [56,57,58,59,60,61].

Under control conditions (24 °C), several HSP60 and constitutive-type HSP70 genes exhibited relatively stable expression, consistent with their proposed housekeeping roles in protein homeostasis [56,57,58,59,60,61]. In contrast, exposure to 44 °C triggered marked upregulation of specific HSPs-including certain sHSPs (e.g., *RpedHsp15.5-3*, *RpedHsp11.1-1*), inducible HSP70 isoforms (*RpedHsp20.8*, *RpedHsp30.8*), and select HSP90 members (*RpedHsp16.9-1*)-many of which showed minimal or no expression at lower temperatures. This strong induction aligns with the severe physiological impact observed at this temperature [10], and suggests these genes may be closely associated with the organism’s response to extreme thermal stress.

Notably, sex-specific differences were evident in HSP expression profiles. At 44 °C, females generally displayed higher induction levels of multiple stress-responsive HSPs, including several sHSPs and HSP70s, whereas a canonical cytosolic HSP90 homolog (*RpedHsp83.6*) was predominantly expressed in males. These contrasting patterns echo previously reported sex differences in thermal physiology [10] and hint at divergent regulatory strategies between the sexes, though the underlying mechanisms remain to be elucidated. The HSP40 family also exhibited diverse expression behaviors. Most members, such as *RpedHsp42.0-1* and *RpedHsp40.5-2*, showed gradual upregulation with increasing temperature, consistent with their role as co-chaperones of HSP70. However, some displayed sex- or temperature-biased expression—for example, *RpedHsp14.3* was strongly activated in females at 44 °C, while *RpedHsp44.8-1* was nearly undetectable in males under the same condition. Additionally, certain HSP40 genes (e.g., *RpedHsp37.7-2/3*) peaked at 40 °C, suggesting functional differentiation among family members in responding to varying degrees of thermal stress.

In summary, this study identifies a suite of HSP genes—particularly *RpedHsp15.5-3*, *RpedHsp30.8*, and *RpedHsp16.9-1*—that are specifically and robustly induced under lethal high temperatures. These candidates warrant further investigation and may serve as useful molecular indicators of thermal stress in *R. pedestris*. Compared with prior transcriptomic studies on other soybean-associated insects which focused on RNAi-related genes in *Leguminivora glycinivorella* [62], host adaptation in *Aphis glycines* [63], or insecticide responses in *Thrips flavus* [64], none systematically characterized the full HSP repertoire or its sex-specific expression under physiologically defined thermal stress. Our findings enhance the understanding of the transcriptional architecture of the heat shock response in this key soybean pest and provide a valuable reference for future comparative studies on other insect species associated with soybean agroecosystems, such as *L. glycinivorella* [55], *A. glycines* [56], *Orosius orientalis* [12], and *T. flavus* [57]. Nevertheless, this study is limited to transcriptomic inference under acute, fixed-temperature exposures, without functional validation or phenotypic correlation. Future work should therefore validate the thermoprotective roles of key candidates—such as *RpedHsp15.5-3*, *RpedHsp30.8*, and male-specific *RpedHsp83.6*—using RNAi or gene editing, and assess their impacts on sex-specific fitness under ecologically realistic thermal regimes Such functional characterization will be essential to evaluate their potential relevance to pest management strategies.

## 5. Conclusions

This study demonstrates that *R. pedestris* exhibits significant sex-specific differences in thermal tolerance, with females showing higher lethal thresholds than males (HLT50: 39.76 °C vs. 38.45 °C; HLT90: 42.99 °C vs. 42.44 °C). Transcriptomic profiling across physiologically relevant temperatures (24 °C, 40 °C, and 44 °C) revealed a multi-layered heat shock protein response characterized by subfamily diversification and sexual dimorphism. Females preferentially upregulated multiple stress-responsive HSPs such as *RpedHsp15.5-3* and *RpedHsp30.8*, whereas males specifically induced *RpedHsp83.6*. Notably, *RpedHsp15.5-3*, *RpedHsp30.8*, and *RpedHsp16.9-1* were strongly and selectively activated under lethal heat (44 °C), suggesting their utility as molecular indicators of extreme thermal stress. These findings establish a foundation for future research into the molecular mechanisms underlying sex-biased thermotolerance in *R. pedestris*.

## Figures and Tables

**Table 1:** Fitting equations for survival rate curves and Temperature zones of *R. pedestris* under different temperature and time treatments.

Sex	Regression Equation	R^2^	Optimal Temperature Zone (°C)	Zone of Sublethal Temperature (°C)	High Fatal Temperature Zone (°C)
♀ ^a^	$y=\frac{105.47}{1+e^{\left(23.00-0.58x\right)}}$	0.99	<34.73	34.73~43.77	>43.77
♂ ^b^	$y=\frac{110.06}{1+e^{\left(16.44-0.42x\right)}}$	0.98	<31.68	32.14~43.24	>43.24

**Note**: ^a^ Represents female, ^b^ Represents male.

## Data Availability

The original contributions presented in this study are included in the article/Supplementary Materials. Further inquiries can be directed to the corresponding authors.

## Supplementary Materials

The following supporting information can be downloaded at: https://www.mdpi.com/article/10.3390/biology15070552/s1, Table S1: Primers for *R. pedestris* actin and upregulated HSP genes; Table S2: Analysis of differences in survival rates between males and females; Table S3: Confidence interval of regression equations of *R. pedestris*. Table S4: Transcriptome assembly summary of *R. pedestris*; Table S5: Assembly of unigenes and transcripts of *R. pedestris*; Table S6: Annotated of *R. pedestris* with BLAST; Table S7: Top differentially expressed genes in *R. pedestris* under heat stress across sexes; Table S8: The information of Hsp gene superfamily in *R. pedestris*; Date S1: Sequence of the RpedHSPs.
