# Supplementary material for: Sex-Specific Thermal Adaptation in Riptortus pedestris: Integrating Logistic Thresholds and Transcriptomic Responses
Source: Biology (Basel). 2026 Mar 30;15(7):552. doi: 10.3390/biology15070552 (PMC13072309; doi:10.3390/biology15070552)
Supplement: Supplementary file 1 [file biology-15-00552-s001.zip › biology-4180609-supplementary.pdf]

Supplementary Materials

Table S1 Primers for *R. pedestris* actin and upregulated HSP genes

Gene-id	Primers sequences
RPL7A-F	CTAAGATCAAGCAGAAGGATG
RPL7A-R	TGGCAGGAACAGAACAAG
RpedHsp15.5-3F	CGCCCATGGAGGAATCTGTT
RpedHsp15.5-3R	TCACCTTGAGCTCTTCAGGC
RpedHsp20.8-F	TCCCATTGGCATCGAGATCG
RpedHsp20.8-R	TTCGAAGGTGAGCGAGCAAT
RpedHsp14.4-1-F	TCCCATTGGCATCGAGATCG
RpedHsp14.4-1-R	TTCGAAGGTGAGCGAGCAAT
RpedHsp30.8-F	ATCGAAATCTTCACCGCCGA
RpedHsp30.8-R	TGGGCGGAGGTACTTTTGAC
RpedHsp11.1-1-F	CACCTTCAGCTCTTCCGGTT
RpedHsp11.1-1-R	TGCGACCCTGGAGGAATCTA
RpedHsp9.8-F	GTCTGAGTTTGCTTGCACGG
RpedHsp9.8-R	TCTTGGTTGACGTAGCTCCG
RpedHsp14.1-F	CAATGAGCCGTTTAGCGTCG
RpedHsp14.1-R	TAGCTACGTGGCCTTTTCGG
RpedHsp4.9-1-F	CAAATGGGTTCCTCCTGTAGTCA
RpedHsp4.9-1-R	GTTAGTCGGCAAGCATCTTGAAG
RpedHsp5.4-1-F	GATGGGAACTTCCAGAGGATGAA
RpedHsp5.4-1-R	AGAATCTACAAGCCTGTTGCCTA
RpedHsp4.6-1-F	GGAACTCCTTGATGAACTTGCTG
RpedHsp4.6-1-R	TGTGTGGTACCTCAGAAGTTCTG
RpedHsp16.9-1-F	TACATGGCAGCTAAGAAACACCT
RpedHsp16.9-1-R	ATCCTTCACAGCCTTGTCATTCT
RpedHsp8.2-2-F	CAGATACCCACTTCGAAGAGGAG
RpedHsp8.2-2-R	ACAACCTAGACTTGCCGATCTTT
RpedHsp6.3-F	CCGACACTTGCTGATCTCTATGA
RpedHsp6.3-R	CTCAGGTATCCAGATCGAAGTGG[1]

[1] Primer sequences, amplicon sizes, and amplification efficiencies are based on validation data reported in Wang et al. (2023) [1]

[1] Wang, L.; Liu, Q.; Guo, P.; Gao, Z.; Chen, D.; Zhang, T.; Ning, J. Evaluation of Reference Genes for Quantitative Real-Time PCR Analysis in the Bean Bug, *Riptortus pedestris* (Hemiptera: Alydidae). Insects 2023, 14, 960. https://doi.org/10.3390/insects14120960

Table S2 Analysis of differences in survival rates between males and females

Temperature (°C)	Time (hours)	Z value	Asymptotic significance (double tailed)
24	1	0	1
24	2	0	1
24	3	0	1
24	4	0	1
32	1	-1.581	0.114
32	2	0	1
32	3	-1	0.317
32	4	-1	0.317
36	1	0	1
36	2	-1.581	0.114
36	3	-2.023	0.043
36	4	-2.023	0.043
40	1	-0.745	0.456
40	2	-1	0.317
40	3	-1	0.317
40	4	-2.121	0.034
42	1	-1	0.317
42	2	-2.023	0.043
42	3	-2.236	0.025
42	4	-2.023	0.043
44	1	-1.826	0.068
44	2	-2.023	0.043
44	3	0	1
44	4	-1	0.317

Table S3 Confidence interval of regression equations

Lower limit of 95% CI - Female	Upper limit of 95% CI - Female	Lower limit of 95% CI - Male	Upper limit of 95% CI - Male
0.6188	1.0000	0.9711	0.9711
0.9183	0.9183	0.9033	0.9033
0.4843	1.0000	0.4688	1.0000
0.4171	1.0000	0.7678	0.7678
0.4721	0.9612	0.4555	0.9445
0.9856	0.9856	0.9711	0.9711
0.9183	0.9183	0.5365	1.0000
0.4843	1.0000	0.8356	0.8356
0.4171	1.0000	0.4010	1.0000
0.4721	0.9612	0.4555	0.9445
0.9856	0.9856	0.6043	1.0000
0.9183	0.9183	0.5365	1.0000
0.8511	0.8511	0.4688	1.0000
0.7839	0.7839	0.7678	0.7678
0.4721	0.9612	0.4555	0.9445
0.6188	1.0000	0.9711	0.9711
0.5515	1.0000	0.5365	1.0000
0.8511	0.8511	0.4688	1.0000
0.4171	1.0000	0.4010	1.0000
0.4721	0.9612	0.7000	0.7000

Table S4 Transcriptome assembly summary of *R. pedestris*

Sample name	Read Number(bp)	Base Number(bp)	GC(%)	%≥Q30
A	59921308	8988196200	46.67%	91.54%
B	56488886	8473332900	46.56%	90.92%
C	55664920	8349738000	46.66%	91.02%
D	53196692	7979503800	47.12%	90.95%
E	52361186	7854177900	45.28%	91.67%
F	47437106	7115565900	46.30%	88.85%

Table S5 Assembly of unigenes and transcripts of *R. pedestris*

Length Range	Transcript	Unigene
500-1000	133278	68210
1000+	52664	23475
Total Number	443995	270199
Total Length	244280162	131166405
N50 Length	720	575
Mean Length	550.19	485.44

Table S6 Annotated of *R. pedestris* with BLAST

Anno database	Annotated Number	length<300	300<=length<1000	length>=1000
Annotated in CDD	28497	7429	12698	8370
Annotated in PFAM	32261	7417	14649	10195
Annotated in KEGG	27601	9533	12445	5623
Annotated in KOG	41804	11145	19043	11616
Annotated in Swissprot	71269	24910	31944	14415
Annotated in GO	54683	20718	23673	10292
Annotated in NR	81356	27129	37930	16297
Annotated in NT	99470	50881	38631	9958

Table S7 Top differentially expressed genes in *R. pedestris* under heat stress across sexes

Gene id	Description	Scientific Name
**24♀vs24♂up**		
TRINITY_DN138969_c0_g1	uncharacterized protein	Halyomorpha halys
TRINITY_DN139062_c6_g1	vitellogenin	Riptortus clavatus
TRINITY_DN140407_c0_g1	cyanoprotein alpha subunit precursor	Riptortus clavatus
TRINITY_DN140143_c1_g2	unkown protein, partial	Riptortus pedestris
TRINITY_DN128604_c1_g1	gpi-anchor transamidase, partial	Riptortus pedestris
TRINITY_DN130104_c2_g1	hypothetical protein, partial	Riptortus pedestris
TRINITY_DN132159_c0_g2	unnamed protein product	Nezara viridula
TRINITY_DN137595_c0_g2	carboxypeptidase and/or zinc-ion binding protein, partial	Riptortus pedestris
TRINITY_DN126273_c0_g1	hypothetical protein, partial	Riptortus pedestris
TRINITY_DN130309_c0_g1	uncharacterized protein	Halyomorpha halys
**24♀vs24♂ down**		
TRINITY_DN139244_c0_g2	tubulin alpha chain	Halyomorpha halys
TRINITY_DN128975_c0_g1	cytosol aminopeptidase	Halyomorpha halys
TRINITY_DN140422_c1_g3	tubulin beta-4B chain	Halyomorpha halys
TRINITY_DN142213_c1_g1	Hypothetical protein GE061_019586	Apolygus lucorum
TRINITY_DN139244_c0_g4	tubulin alpha chain	Halyomorpha halys
TRINITY_DN137943_c0_g2	sarcoplasmic reticulum histidine-rich calcium-binding protein	Halyomorpha halys
TRINITY_DN124068_c0_g1	Hypothetical protein O3M35_000928	Rhynocoris fuscipes
TRINITY_DN135536_c0_g1	cytosol aminopeptidase	Halyomorpha halys
TRINITY_DN138047_c0_g1	cytosol aminopeptidase	Halyomorpha halys

TRINITY_DN140819_c0_g4	cytosol aminopeptidase	Halyomorpha halys
**40♀vs40♂ up**		
TRINITY_DN138553_c0_g1	gpi-anchor transamidase, partial	Riptortus pedestris
TRINITY_DN133110_c1_g1	unknown secreted protein	Riptortus pedestris
TRINITY_DN141476_c1_g2	hypothetical protein JTB14_021408	Gonioctena quinquepunctata
TRINITY_DN129603_c0_g1	unnamed protein product	Nezara viridula
TRINITY_DN130309_c0_g1	uncharacterized protein	Halyomorpha halys
TRINITY_DN135052_c0_g3	cytoplasmic polyadenylation element-binding protein isoform X1	Halyomorpha halys
TRINITY_DN142542_c1_g1	divergent protein kinase domain 1C	Halyomorpha halys
TRINITY_DN123063_c0_g1	uncharacterized protein	Halyomorpha halys
TRINITY_DN141461_c4_g1	transcription factor MafA	Halyomorpha halys
TRINITY_DN118183_c0_g1	glucose dehydrogenase [FAD, quinone]	Halyomorpha halys
**40♀vs40♂ down**		
TRINITY_DN128975_c0_g1	cytosol aminopeptidase	Halyomorpha halys
TRINITY_DN141236_c0_g1	hypothetical protein L9F63_005582, partial	Diploptera punctata
TRINITY_DN136012_c0_g2	hypothetical protein O3M35_012580	Rhynocoris fuscipes
TRINITY_DN140422_c1_g3	tubulin beta-4B chain	Halyomorpha halys
TRINITY_DN142213_c1_g1	hypothetical protein GE061_019586	Apolygus lucorum
TRINITY_DN140201_c0_g2	unnamed protein product r	Nezara viridula
TRINITY_DN140819_c0_g3	cytosol aminopeptidase	Halyomorpha halys
TRINITY_DN137943_c0_g2	sarcoplasmic reticulum histidine-rich calcium-binding protein	Halyomorpha halys
TRINITY_DN124068_c0_g1	hypothetical protein O3M35_000928	Rhynocoris fuscipes
TRINITY_DN135536_c0_g1	cytosol aminopeptidase	Halyomorpha halys

**44♀vs44♂ up**		
TRINITY_DN129313_c0_g1	unnamed protein product	Nezara viridula
TRINITY_DN122459_c0_g1	unnamed protein product	Nezara viridula
TRINITY_DN125535_c0_g1	vasa, partial	Oncopeltus fasciatus
TRINITY_DN131062_c0_g1	unnamed protein product	Nezara viridula
TRINITY_DN130309_c0_g1	uncharacterized protein	Halyomorpha halys
TRINITY_DN139863_c0_g1	uncharacterized protein YdcI	Halyomorpha halys
TRINITY_DN137287_c1_g1	E3 ubiquitin-protein ligase SIAH1 isoform X2	Halyomorpha halys
TRINITY_DN137613_c1_g2	hypothetical protein	Riptortus pedestris
TRINITY_DN114144_c0_g1	unnamed protein product	Nezara viridula
TRINITY_DN128096_c0_g1	nucleolar GTP-binding protein	Riptortus pedestris
**44♀vs44♂ down**		
TRINITY_DN128975_c0_g1	cytosol aminopeptidase	Halyomorpha halys
TRINITY_DN140422_c1_g3	tubulin beta-4B chain	Halyomorpha halys
TRINITY_DN139244_c0_g4	tubulin alpha chain	Halyomorpha halys
TRINITY_DN137943_c0_g2	sarcoplasmic reticulum histidine-rich calcium-binding protein	Halyomorpha halys
TRINITY_DN135536_c0_g1	cytosol aminopeptidase	Halyomorpha halys
TRINITY_DN138047_c0_g1	cytosol aminopeptidase	Halyomorpha halys
TRINITY_DN140819_c0_g4	cytosol aminopeptidase	Halyomorpha halys
TRINITY_DN128975_c0_g2	unnamed protein product	Nezara viridula
TRINITY_DN143461_c8_g1	protein furry isoform X1	Halyomorpha halys
TRINITY_DN129267_c0_g1	calmodulin-like protein 5	Halyomorpha halys
TRINITY_DN140422_c1_g6	tubulin beta-4B chain	Halyomorpha halys

**24♀vs40♀ up**		
TRINITY_DN139386_c1_g2	hypothetical protein, partial	Riptortus pedestris
TRINITY_DN140014_c0_g1	hypothetical protein GE061_015263	Apolygus lucorum
TRINITY_DN143335_c4_g2	unnamed protein product	Nezara viridula
TRINITY_DN134790_c0_g2	clathrin coat associated protein ap-50	Riptortus pedestris
TRINITY_DN138752_c4_g1	heat shock protein, partial	Bemisia tabaci
TRINITY_DN138752_c4_g5	hypothetical protein, partial	Riptortus pedestris
TRINITY_DN138752_c4_g4	heat shock 70 kDa protein-like, partial	Halyomorpha halys
TRINITY_DN109796_c0_g1	unnamed protein product	Nezara viridula
TRINITY_DN140143_c1_g4	unkown protein, partial	Riptortus pedestris
TRINITY_DN137970_c0_g1	hypothetical protein	Riptortus pedestris
**24♀vs40♀ down**		
TRINITY_DN132159_c0_g2	unnamed protein product	Nezara viridula
TRINITY_DN119648_c0_g2	synaptotagmin-16 isoform X2	Halyomorpha halys
TRINITY_DN139500_c2_g1	unknown secreted protein	Riptortus pedestris
TRINITY_DN130170_c0_g4	unkown protein, partial	Riptortus pedestris
TRINITY_DN143035_c3_g4	LOW QUALITY PROTEIN: heat shock 70 kDa protein-like	Bactrocera neohumeralis
TRINITY_DN139087_c1_g1	late secretory pathway protein AVL9 homolog	Halyomorpha halys
TRINITY_DN83080_c0_g1	Rho GTPase protein rac1	Cotesia glomerata
TRINITY_DN3556_c0_g1	ribulose bisphosphate carboxylase small chain, chloroplastic	Prunus dulcis
TRINITY_DN3556_c0_g1	uncharacterized protein LOC112211718	Halyomorpha halys
TRINITY_DN134631_c0_g1	unnamed protein product	Nezara viridula
**24♀vs44♀ up**		

TRINITY_DN139386_c1_g2	hypothetical protein, partial	Riptortus pedestris
TRINITY_DN143252_c3_g4	amino acid permease	Trypanosoma grayi
TRINITY_DN131166_c8_g1	hypothetical protein, partial	Riptortus pedestris
TRINITY_DN89327_c0_g1	unkown protein	Riptortus pedestris
TRINITY_DN143252_c3_g2	amino acid permease	Trypanosoma grayi
TRINITY_DN131166_c3_g3	heat shock protein 68-like	Bactrocera oleae
TRINITY_DN140887_c1_g3	protein lethal(2)essential for life isoform X2	Halyomorpha halys
TRINITY_DN132604_c3_g3	unnamed protein product	Nezara viridula
TRINITY_DN110250_c0_g1	unnamed protein product	Allacma fusca
TRINITY_DN123674_c0_g3	70 kDa heat shock protein, partial	Pyrrhocoris apterus
**24♀vs44♀ down**		
TRINITY_DN132159_c0_g2	unnamed protein product	Nezara viridula
TRINITY_DN132076_c0_g1	uncharacterized protein	Halyomorpha halys
TRINITY_DN38569_c0_g1	hypothetical protein PRUPE_7G068100	Prunus persica
TRINITY_DN139223_c0_g2	unkown protein, partial	Riptortus pedestris
TRINITY_DN143304_c6_g4	unkown protein, partial	Riptortus pedestris
TRINITY_DN143404_c4_g2	low-density lipoprotein receptor-related protein 1-like	Halyomorpha halys
TRINITY_DN132967_c2_g3	troponin I 2 isoform X1	Halyomorpha halys
TRINITY_DN131430_c0_g1	protein vestigial	Halyomorpha halys
TRINITY_DN140761_c1_g5	homeobox protein Mohawk isoform X2	Halyomorpha halys
TRINITY_DN143304_c6_g3	unkown protein	Riptortus pedestris
**24♂vs40♂ up**		
TRINITY_DN139386_c1_g2	hypothetical protein, partial	Riptortus pedestris

TRINITY_DN139217_c0_g2	cathepsin B	Riptortus pedestris
TRINITY_DN143152_c4_g3	MTOR-associated protein MEAK7	Halyomorpha halys
TRINITY_DN138752_c4_g5	hypothetical protein, partial	Riptortus pedestris
TRINITY_DN138752_c4_g4	heat shock 70 kDa protein-like, partial	Halyomorpha halys
TRINITY_DN140887_c1_g3	protein lethal(2)essential for life isoform X2	Halyomorpha halys
TRINITY_DN138606_c1_g1	uncharacterized protein LOC106685943	Halyomorpha halys
TRINITY_DN132173_c0_g1	unknown secreted protein	Riptortus pedestris
TRINITY_DN131060_c1_g2	ribosomal protein S20	Riptortus pedestris
TRINITY_DN132165_c1_g1	unnamed protein product	Nezara viridula
**24♂vs40♂ down**		
TRINITY_DN138147_c1_g1	thanatin	Riptortus pedestris
TRINITY_DN132159_c0_g2	unnamed protein product	Nezara viridula
TRINITY_DN140210_c4_g2	unnamed protein product	Nezara viridula
TRINITY_DN138147_c1_g2	thanatin	Riptortus pedestris
TRINITY_DN132076_c0_g1	uncharacterized protein	Halyomorpha halys
TRINITY_DN139223_c0_g2	unkown protein, partial	Riptortus pedestris
TRINITY_DN143304_c6_g4	unkown protein, partial	Riptortus pedestris
TRINITY_DN131944_c0_g2	hypothetical protein AAG570_014155	Ranatra chinensis
TRINITY_DN140761_c1_g5	homeobox protein Mohawk isoform X2	Halyomorpha halys
TRINITY_DN143304_c6_g3	unkown protein	Riptortus pedestris
**24♂vs44♂ up**		
TRINITY_DN130104_c2_g1	hypothetical protein, partial	Riptortus pedestris
TRINITY_DN134046_c0_g3	cyanoprotein alpha subunit precursor	Riptortus clavatus

TRINITY_DN133050_c2_g3	lysophosphatidic acid receptor 3	Halyomorpha halys
TRINITY_DN126273_c0_g1	hypothetical protein, partial	Riptortus pedestris
TRINITY_DN135259_c0_g1	cytochromeP450	Riptortus pedestris
TRINITY_DN131166_c2_g8	hypothetical protein, partial	Riptortus pedestris
TRINITY_DN128257_c6_g1	heat shock protein 70 A1-like	Photinus pyralis
TRINITY_DN137107_c2_g1	cysteine rich secreted protein	Riptortus pedestris
TRINITY_DN140385_c0_g2	cathepsin B	Riptortus pedestris
TRINITY_DN137976_c5_g1	unkown protein	Riptortus pedestris
**24♂vs44♂ down**		
TRINITY_DN139217_c0_g2	cathepsin B	Riptortus pedestris
TRINITY_DN130574_c0_g6	rhodanese domain-containing protein CG4456-like isoform X4	Halyomorpha halys
TRINITY_DN133448_c0_g2	unknown secreted protein, partial	Riptortus pedestris
TRINITY_DN143097_c2_g1	Hypothetical protein CINCED_3A014223, partial	Cinara cedri
TRINITY_DN128500_c5_g1	uncharacterized protein LOC126767096	Bactrocera neohumeralis
TRINITY_DN132915_c1_g1	unknown secreted protein, partial	Riptortus pedestris
TRINITY_DN137364_c2_g1	hypothetical protein J6590_012189	Homalodisca vitripennis
TRINITY_DN136625_c0_g1	uncharacterized protein	Halyomorpha halys
TRINITY_DN129378_c4_g1	hypothetical protein O0L34_g18776	Tuta absoluta
TRINITY_DN125931_c7_g1	AAEL007848-PA, partial	Aedes aegypti
**40♀vs44♀ up**		
TRINITY_DN133433_c1_g4	sulphate transporter	Riptortus pedestris
TRINITY_DN143159_c2_g4	sodium-coupled monocarboxylate transporter 1-like isoform X2	Rhodnius prolixus
TRINITY_DN134046_c0_g3	cyanoprotein alpha subunit precursor	Riptortus clavatus

TRINITY_DN136649_c0_g2	horizontal transferred salivary protein	Riptortus pedestris
TRINITY_DN137532_c1_g2	gamma-glutamyl hydrolase, putative	Riptortus pedestris
TRINITY_DN123872_c0_g1	sulfotransferase	Riptortus pedestris
TRINITY_DN141704_c2_g1	unnamed protein product	Nezara viridula
TRINITY_DN137055_c0_g1	thanatin	Riptortus pedestris
TRINITY_DN142881_c2_g1	unnamed protein product	Nezara viridula
TRINITY_DN122697_c0_g1	heat shock protein cognate 3	Riptortus pedestris
**40♀vs44♀ down**		
TRINITY_DN139217_c0_g2	cathepsin B	Riptortus pedestris
TRINITY_DN129650_c1_g2	unkown protein, partial	Riptortus pedestris
TRINITY_DN143578_c9_g1	ABC transporter G family member 23-like	Rhodnius prolixus
TRINITY_DN131903_c1_g1	unkown protein	Riptortus pedestris
TRINITY_DN136816_c1_g1	endocuticle structural glycoprotein SgAbd-2-like isoform X1	Halyomorpha halys
TRINITY_DN139223_c0_g2	unkown protein, partial	Riptortus pedestris
TRINITY_DN130040_c2_g1	unkown protein	Riptortus pedestris
TRINITY_DN133050_c2_g2	cathepsin B	Riptortus pedestris
TRINITY_DN142263_c3_g1	unknown secreted protein	Riptortus pedestris
TRINITY_DN143180_c1_g2	uncharacterized protein	Halyomorpha halys
**40♂vs44♂ up**		
TRINITY_DN121563_c0_g1	facilitated trehalose transporter Tret1	Halyomorpha halys
TRINITY_DN139911_c0_g1	unnamed protein product	Nezara viridula
TRINITY_DN137219_c0_g3	venom serine protease isoform X2	Halyomorpha halys
TRINITY_DN131882_c1_g1	chemosensory protein CSP3	Spodoptera exigua

TRINITY_DN138147_c1_g2	thanatin	Riptortus pedestris
TRINITY_DN142519_c0_g2	unnamed protein product	Nezara viridula
TRINITY_DN127392_c0_g2	facilitated trehalose transporter Tret1	Halyomorpha halys
TRINITY_DN143159_c2_g4	sodium-coupled monocarboxylate transporter 2	Halyomorpha halys
TRINITY_DN142279_c0_g3	synaptic vesicle glycoprotein 2B isoform X1	Halyomorpha halys
TRINITY_DN124260_c1_g1	unnamed protein product	Nezara viridula
**40♂vs44♂ down**		
TRINITY_DN128970_c1_g1	sugar transporter	Riptortus pedestris
TRINITY_DN140210_c4_g2	unnamed protein product	Nezara viridula
TRINITY_DN143455_c8_g4	unkown protein	Riptortus pedestris
TRINITY_DN137970_c0_g2	hypothetical protein	Riptortus pedestris
TRINITY_DN131903_c1_g1	unkown protein	Riptortus pedestris
TRINITY_DN139223_c0_g2	unkown protein, partial	Riptortus pedestris
TRINITY_DN139833_c1_g1	putative oxidoreductase GLYR1 homolog	Halyomorpha halys
TRINITY_DN130838_c1_g1	unnamed protein product	Allacma fusca
TRINITY_DN143240_c4_g1	unkown protein, partial	Riptortus pedestris
TRINITY_DN137970_c0_g1	hypothetical protein	Riptortus pedestris

Table S8 The information of Hsp gene superfamily in *Riptortus pedestris*.

Gene Identifier	Family	Gene Name	CDS	Mw (kDa)	pI	Subcelluar Location	Strand
TRINITY_DN140887_c0_g2	sHsp	RpedHsp15.5-3	1-240	15.5	5.58	Cytoplasmic/Mitochondrial	minus
TRINITY_DN140887_c1_g3	sHsp	RpedHsp11.1-1	3-299	11.1	4.72	Extracellular	minus
TRINITY_DN140215_c1_g1	sHsp	RpedHsp4.3-1	91-210	4.3	10.29	Cytoplasmic	plus
TRINITY_DN133666_c0_g1	Hsp40	RpedHsp56.5	256-1737	56.5	6.11	Cytoplasmic/Periplasmic	plus
TRINITY_DN130223_c0_g1	Hsp40	RpedHsp40.5-2	153-1226	40.5	5.92	Cytoplasmic	plus
TRINITY_DN136753_c1_g2	Hsp40	RpedHsp44.8-1	18-1378	44.8	6.65	Cytoplasmic	plus
TRINITY_DN122823_c0_g1	Hsp40	RpedHsp37.7-2	36-1049	37.7	8.9	Cytoplasmic	minus
TRINITY_DN133727_c0_g2	Hsp40	RpedHsp14.3	3-416	14.3	6.74	Periplasmic/Cytoplasmic	minus
TRINITY_DN125048_c0_g1	Hsp40	RpedHsp42.0-1	67-1179	42	5.71	Cytoplasmic	plus
TRINITY_DN134604_c1_g1	Hsp40	RpedHsp42.6	134-1279	42.6	7.32	Cytoplasmic	plus
TRINITY_DN139010_c1_g3	Hsp40	RpedHsp37.7-3	118-1137	37.7	9.2	Cytoplasmic	plus
TRINITY_DN140233_c0_g2	Hsp60	RpedHsp17.7-1	55-546	17.7	9.95	Cytoplasmic	plus
TRINITY_DN140915_c0_g1	Hsp60	RpedHsp11.2-1	321-635	11.2	9.41	Periplasmic/Cytoplasmic	plus
TRINITY_DN140233_c0_g1	Hsp60	RpedHsp25.1	27-718	25.1	5.87	Cytoplasmic	plus
TRINITY_DN140915_c0_g5	Hsp60	RpedHsp7.4-3	5-226	7.4	4.39	Cytoplasmic/Periplasmic	plus
TRINITY_DN140915_c0_g3	Hsp60	RpedHsp35.0-1	82-1059	35	5.24	Cytoplasmic	plus
TRINITY_DN139396_c0_g2	Hsp60	RpedHsp37.7-1	178-1227	37.7	4.86	Cytoplasmic	plus
TRINITY_DN140667_c1_g2	Hsp60	RpedHsp24.6-2	247-924	24.6	9.44	Cytoplasmic	plus

TRINITY_DN140667_c1_g3	Hsp60	RpedHsp17.0-1	193-666	17	5.34	Cytoplasmic	plus
TRINITY_DN140667_c1_g1	Hsp60	RpedHsp11.2-4	34-354	11.2	4.62	Cytoplasmic	plus
TRINITY_DN128235_c0_g3	Hsp60	RpedHsp59.5	188-1813	59.5	5.76	Cytoplasmic	minus
TRINITY_DN140233_c0_g4	Hsp60	RpedHsp7.8-1	71-304	7.8	4.38	Periplasmic/Cytoplasmic	plus
TRINITY_DN139396_c0_g1	Hsp60	RpedHsp7.7-2	126-335	7.7	10.35	Cytoplasmic	plus
TRINITY_DN134971_c1_g4	Hsp70	RpedHsp6.6-1	3-191	6.6	9.16	Periplasmic/Cytoplasmic	minus
TRINITY_DN142185_c3_g1	Hsp70	RpedHsp18.4	2-487	18.4	8.64	Cytoplasmic	minus
TRINITY_DN138982_c0_g1	Hsp70	RpedHsp29.9	404-1216	29.9	5.33	Cytoplasmic/Periplasmic	minus
TRINITY_DN142368_c2_g1	Hsp70	RpedHsp74.9	3-2000	74.9	6.47	Cytoplasmic	minus
TRINITY_DN135093_c0_g1	Hsp70	RpedHsp5.4-2	45-194	5.4	11.6	Cytoplasmic	plus
TRINITY_DN138752_c4_g3	Hsp70	RpedHsp9.8	3-278	9.8	4.34	Periplasmic/Cytoplasmic	minus
TRINITY_DN142185_c3_g2	Hsp70	RpedHsp13.9-1	3-380	13.9	6.72	Cytoplasmic/Periplasmic	minus
TRINITY_DN142185_c1_g3	Hsp70	RpedHsp8.6-1	3-227	8.6	6.15	Cytoplasmic	minus
TRINITY_DN140061_c0_g2	Hsp70	RpedHsp35.4	139-1116	35.4	5.35	Cytoplasmic	plus
TRINITY_DN142123_c1_g1	Hsp70	RpedHsp4.2-1	101-205	4.2	9.18	Cytoplasmic	plus
TRINITY_DN138000_c1_g1	Hsp70	RpedHsp29.7	2-799	29.7	9.71	OuterMembrane/Cytoplasmic	minus
TRINITY_DN142751_c2_g3	Hsp70	RpedHsp11.5-1	1-315	11.5	5.1	Cytoplasmic/Periplasmic	minus
TRINITY_DN134971_c1_g3	Hsp70	RpedHsp8.9-2	1-246	8.9	4.65	Cytoplasmic	minus
TRINITY_DN138752_c4_g5	Hsp70	RpedHsp14.1	3-383	14.1	8.59	Periplasmic/Cytoplasmic	minus
TRINITY_DN122697_c0_g1	Hsp70	RpedHsp7.4-4	254-451	7.4	4.95	Cytoplasmic	plus
TRINITY_DN139386_c1_g2	Hsp70	RpedHsp30.8	1-837	30.8	8.71	Cytoplasmic	minus
TRINITY_DN140128_c0_g1	Hsp70	RpedHsp9.2-2	1-240	9.2	9.22	Cytoplasmic	minus

TRINITY_DN142123_c3_g2	Hsp70	RpedHsp8.9-3	3-245	8.9	4.81	Cytoplasmic	minus
TRINITY_DN139386_c0_g1	Hsp70	RpedHsp69.6	536-2416	69.6	6.07	OuterMembrane	minus
TRINITY_DN142123_c3_g3	Hsp70	RpedHsp46.1	2-1258	46.1	7.68	Cytoplasmic	minus
TRINITY_DN140128_c0_g2	Hsp70	RpedHsp34.5	296-1246	34.5	5.19	Periplasmic/Cytoplasmic	minus
TRINITY_DN141591_c1_g1	Hsp70	RpedHsp88.8	390-2777	88.8	5.2	Cytoplasmic	minus
TRINITY_DN134971_c1_g6	Hsp70	RpedHsp30.3	70-891	30.3	4.91	Periplasmic	minus
TRINITY_DN141014_c0_g3	Hsp70	RpedHsp14.0-3	155-541	14	5.01	OuterMembrane	plus
TRINITY_DN141014_c0_g1	Hsp70	RpedHsp13.9-2	172-543	13.9	8.73	Cytoplasmic	plus
TRINITY_DN138355_c2_g1	Hsp70	RpedHsp81.8	19-2224	81.8	6.03	OuterMembrane	plus
TRINITY_DN142185_c3_g3	Hsp70	RpedHsp18.1	3-479	18.1	5.05	Cytoplasmic	minus
TRINITY_DN123674_c0_g3	Hsp70	RpedHsp14.4-1	1-387	14.4	5.19	Cytoplasmic	minus
TRINITY_DN131166_c4_g1	Hsp70	RpedHsp20.8	2-571	20.8	4.96	Cytoplasmic/Periplasmic	minus
TRINITY_DN141591_c1_g2	Hsp70	RpedHsp11.1-2	1-297	11.1	8.52	InnerMembrane/Cytoplasmic	minus
TRINITY_DN142185_c2_g1	Hsp70	RpedHsp5.8-1	47-205	5.8	9.38	Cytoplasmic	plus
TRINITY_DN132165_c0_g1	Hsp70	RpedHsp8.2-2	1-219	8.2	4.94	Cytoplasmic/Periplasmic	minus
TRINITY_DN132165_c1_g3	Hsp70	RpedHsp4.2-3	431-535	4.2	5.45	Cytoplasmic	plus
TRINITY_DN139626_c2_g1	Hsp70	RpedHsp6.3	43-210	6.3	5.99	Cytoplasmic/Periplasmic	plus
TRINITY_DN140944_c3_g2	Hsp70	RpedHsp4.6-4	60-185	4.6	5.95	Cytoplasmic	plus
TRINITY_DN140944_c4_g2	Hsp70	RpedHsp4.9-1	3-134	4.9	4.64	Cytoplasmic	minus
TRINITY_DN140302_c5_g6	Hsp90	RpedHsp4.6-1	203-319	4.6	9.22	Cytoplasmic	plus
TRINITY_DN140302_c4_g2	Hsp90	RpedHsp5.4-1	101-241	5.4	8.93	Cytoplasmic	plus
TRINITY_DN126669_c0_g1	Hsp90	RpedHsp16.9-1	12-467	16.9	5.02	Cytoplasmic	plus

TRINITY_DN123167_c0_g2	Hsp90	RpedHsp83.6	67-2250	83.6	4.91	Cytoplasmic	minus
TRINITY_DN142471_c0_g1	Hsp90	RpedHsp37.3	150-1145	37.3	4.67	Cytoplasmic	plus
TRINITY_DN133811_c0_g1	Hsp90	RpedHsp40.0	2-1042	40	5.66	Cytoplasmic/Extracellular	plus
TRINITY_DN131590_c1_g3	Hsf	RpedHsp71.9	191-2122	71.9	6.03	OuterMembrane	minus
TRINITY_DN133491_c0_g1	Hsf	RpedHsp25.3	70-759	25.3	5.56	Cytoplasmic/InnerMembrane	minus

Date S1 Sequence of the RpedHSPs

RpedsHsp:

>RpedHsp4.3-1
MAKTAAAIKKLMPMFDRVLVLRAEAVTKTKGGIVIPEKSQ
>RpedHsp15.5-3
MALLPLLSGVLDDLRYPRFTDIYDQHFGFGLVDDWNLIPTSTLLSAPLRSGYLRPWRNLLA
DDSGVSSIRSDKNELRINLDVQQFKPEELKVKVADGYIVVDGKHEERSDEHGFISRQFTRKY
KIPKGVDEASLVS
>RpedHsp11.1-1
MALLPLLSEILDDYRQPRLADLYDQHFGLGLLDNCNVMPTTAVLAAPLRSGYLRPWRNLL
ADQSGVSTIQSDKNEFRINLDVQQFKPEELKVTVADGFI

RpedHsp40:

>RpedHsp56.5
MAATAECDVVMAATEPELLEDEEAKREAESFKEQGNAYYAKKDYNEAYNYYTKAIDMCP
NNASYYGNRAATLMMLGRFREALGDAQQSVRLDDSFVRGHLREGKCHLSLGNAMAACR
SFQRALELDHKNAQAQQEFKNANAVMEYEKIAEVDFEKRDFRKVVFCMDRALEFAPACH
RFKILKAECLAMLGRYPEAQFVASDILRMDSTNADALYVRGLCLYYEDCIEKAVQFFVQAL
RMAPDHEKACLACRNAKALKAKKEDGNKAFKEGNYKLAYELYTEALGIDPNNIKTNAKL
YCNRGTVNSKLRQLEDAIEDCTNAVKLDDTYIKAYLRRAQCYMDTEQFEEAVRDYEKVYQ
TEKTKEHKQLLKNAQLELKKSKRKDYYKILGVDKNASEDEIKKAYRKRALMHHPDRHSG
ASAEVQKEEEKKFKEVGEAFTILSDPKKKTRYDSGQDLDEEGMNMGDFDANNIFKAFFGG
PGGFSFEASGPGNFYFQFG
>RpedHsp40.5-2
MAPQNLSTFCLLLLYLIGAVIAGRDFYKILGVPRSASIKDIKKAYRKLALQLHPDRNPDDPQ
AQEKFQDLGAAYEVLSDSEKRKQYDTYGEEGLKDGHQSSHGDIFSHFFGDFGFMFGGAPR
QQDRNIPRGSDIIVDLEVTLEEVYAGNFVEVVRNKPVARQAPGKRKCNCRQEMRTTQLGP
GRFQMTQEVVCDECPNVKLVNEERTLEVEIEPGVRDGMEYPFIGEGEPHVDGEPGDLRFRI
KVVKHRIFERRGDDLYTNVTVSLVEALVGFEMDITHLDGHKVHISRDKITRPGAKLWKKGE
GLPNFDNNNIKGSLIITFDVDFPKEQLTEEAKEGIKQLLKQGPVQKVYNGLQGY
>RpedHsp44.8-1
MVKETTYYDVLGVKPNATQEELKKAYRKLALKYHPDKNPNEGEKFKQISQAYEVLADSKK
RELYDKGGEQAIKEGGAGGGFGSPMDIFDMFFGGGGRMQRERRGKNVVHQLSVTLEDLY
NGATRKLALQKNVICDKCEGRGGKKGAVECCPNCRGTGMQIRIHQIGPGMVQQIQSVCM
ECQGHGERISPKDRCKSCNGRKIVREKKILEVHIDKGMKDGQKITFHGEGDQEPGLEPGDII
IVLDQKDHAVFTRRGEDLFMCMDIQLVEALCGFQKPISTLDNRTIVITSHPGQIVKHGDIKC
VLNEGMPIYRRPYEKGRLIIEFKVNFPENGFLSPDKLSLLEKLLPERKEVEETDEMDQVELVD
FDPNQERRRHYNGEAYEDDEHHPRGGVQCQTS
>RpedHsp14.3
MGRGRDFYSILGVSKGASADEIKKAYRKLALKYHPDKNKAAGAEEKFKEVAEAYEVLSDK
KKRDIYDKFGEEGLKGGFGGGGSAPSGGGPPPSGTFTYEFHGDPFATFAQVFGSSDPSDAFS
FMSGGGFPFGGGGGGG

>RpedHsp42.6
MLAGRRKRHVPVLWCVVCLLATVVVCSVPTYATTAVSTTANDDDYYAILGLETEREDATER
QIKTAWRQLSKKHHPDLAGEVERQTYQRIQRAYEVLGDRRKRKVYDILGVDGLRKLEQPQ
QQQAMNPFMQLFGAGAVQPADRGTDVNMLLVVPLEDVYKGAAHSLVFTKNKICRACRG
SGARSPQDVVKCTHCGGHGRVIAHVQLMPGFVQEVQQQCQHCRGQGKRINRYCPVCRG
KGTTRGGVKISVDVEAGVPEGHVITYELEADQQPGQVPGDVLLTVVTAPHPRFERVGDDL
HTTVHITLKEALLGFEKTLTHLDGHEVVLHTDKITQHDERVELADEGLPRHHVPSEHGKLF
VTYKIDMPAALSPQQIELLQHHLE
>RpedHsp37.7-3
MMGKDYYKILGLSKGATTEEIKKAYRQLALKYHPDKNKSPGAEDMFKQVAEAYEVLNDK
KKRELYDKYGEEGLKGGHGLSGSGMGTSSFSFHDPKTTFAQFFGTSDPFDLFDFGPTSSAFD
SFYNDLNIDSDAFSDIGLLGSLRGGPGRSQSFNVRGNNARKDKVQDNPIEHDLYVTLDEILK
GTTKKMKISRRALQADGTTKKEDKLLTVNVKPGWKAGTKITFQREGDQGRNKIPADIVFII
RDKPHPVFKREGSDIRYTAKISLKSALCGTQIEIPTLTGESIPLNLSREIIKPTTVKRIQGHGLPL
PKEPSRKGDLLVSFDIIFPDMLTGSVKDIL
>RpedHsp37.7-2
MGKDYYRVLGLKKGASTEEIKKAYRQLALRYHPDKNKSPGAEEKFKEVAEAYEVLSDQKK
RGIYDQYGEEGLKGAAGVPGGNATYTFHGDPRATFAQFFGTSNPFASFFEFGGSSDPFAGFF
DDDDLSFTEIRTSGPAGQFTGHRIYTGGNPSRIEAEQDPPLEFDLPVTLEEVLRGAKKKMKIS
RKVLQPDGLSRIEDKILTINVKPGWKAGTKITFPQEGDQSLHRTPADVIFVIRDKPHAIFKRD
NSNIIYTAKISLKKALLGTALIVPTLTRESVELNVNEVITPQTTKRISNRGLPFPKDPARYGDLI
VNFDISFPERLSKNQKDILQDLLPN
>RpedHsp42.0-1
MFDLSSFSITLNMGKSLCVYVLLLNLLVLSVLVLSERNFYNILGVSRNANLNQIKKAYRKLA
KELHPDKNTDDPNAASKFADLSAAYEVLSNKEKREKYDKCGEECLKKDGMMGEGMDPF
ASFFGDFGFHFGNGDSRHETRKGANIVMDLYVSLEELYNGNFVEITHNKPVLKPAPGTRRC
NCRQELQTKSLGPGRFQMIQQTVCDECPNVKFVTEDRLLEVEIEAGMQDGQEVKFVAEGE
PHLDGDPGDLVLRIKTQPHKVFERKGDDLYTNVTVSLADALTGFSLEIQHLDGRKLTIQRD
KVTWPGARLRKKGEGMPHYENNNLHGALYITFDVEFPKEGFSEEDEESLKKILKQKSVNN
VYNGLREEL

RpedHsp60:
>RpedHsp17.7-1
MLRLLIGYSLKNSRRSYAKDIRFGHEVRSKMLEGVDILADAVAVTMGPKGRNVIIDQPWGS
PKITKDGVTVAKGVELKEKFQNIGAKIVQAVAENTNKKAGDGTTTATVLARAIAKEGFNRI
SRGANPIEIRKGVMLAVEEAKTHLKSLSKQVTTPEEIAQVAT
>RpedHsp11.2-1
MYRLPTVLKTCASARAVLARGYAKDVRFGPEVRGLMLQGVDILADAVAVTMGPKGRNVIL
EQSWGSPKITKDGVTVAKGVELKDKFQNIGAKLVQDVANNTNEEA
>RpedHsp25.1
MKLDKGYISAYFINTAKGAKVEYEDCMVLISEKKLSSIQSVLPALELINKKKKPLLIIAEDVD
GEALSMLVINRLNIGLNVVAIRAPGFGEQRKVILKDIAIATGATVFGDEVGLRIEDLQEKYLG
GVGEIVVTKEESLLLKGKGVKEDVEKRINEIKLAIETTKSDFEKERLQERLGRLTAGVAVLKV
GGCSEVEVNEKKDRVTDALNATRAAIEEGIVPGGGTALLRCIA

>RpedHsp7.4-3
MGYDALNNEYVNMIDRGIIDPTKVVRTALTAAAGVASLLTTAEAVVTEIPSKDPPLPGGGM
GGMGGMGGMGGMM
>RpedHsp35.0-1
MLAVDAIKDHLKTLSKPVTTPEEIAQVATISANGDTAIGKLISDAMKKVGKEGVITVKDGKT
LEDELEIIEGMKFDRGYISPYFINTTKGAKVEFQDCLLLLSEKKISSVQSIIPALEYANSKRKPL
VIIAEDVDGEALSTLVVNRLKIGLQVAAVKAPGFGDNRKATLQDMAIATGGIVFGDEADM
VKLEDVQPSDLGQVGEIVITKDDTLILKGKGKQADIDKRAQQLRDLIADTTSDYEREKLQER
LARLASGVALLKVGGSSEVEVNEKKDRVNDALNATRAAVEEGIVPGGGTALLRCIAKLKEL
KPANEDQKTGVEIVCK
>RpedHsp37.7-1
MKDSLEIIDGIKIENGYVSQSFVNSKGNKTIFENSLVLLCDMKITSVRSIIYALEAAAKRRRPIV
LVAEDFEEEPILAMIINKLKGCCQVAAVKSPGVGDFRSEVLQDMAVALGGKVYGDEALEEK
GDVINPSYFGTAGEVIITKDETLFLKCQGKKEAIEERAQLIRGLMEQVNNDLDLSKLKERLSR
LICGVAILHVGANNETEMKEKKDRVEDALHATKAALEEGIVPGGGVALLRCISEVKKITAQ
NPDEKAGLDILANALKTPCMMIASNAGADASVVVNKVLNSEIEVGYDARNDKYVNMFE
AGIVDPTKVVKTALEDAAAAASLLTTAEAAVTFVFDGDEIPL
>RpedHsp24.6-2
MMQGLDILADTVALTLGPKGRNVVLEQYKRPPKITKDGVTVSEHIDLKNRFQNIGVKLLQE
VAKNTNKAAGDGTTTATILARAIAKDGLNRLSPNINSNLLRKGIYLALSSIEKKLKEISKPVM
SSDEIAQVATISANGDKEVGKLISDAMYQLGPCRYFSRWSACSKEVKFGGECRQLMMQGL
DILADTVALTLGPKGRNVVLEQYKRPPKITKDGVTVSEHIDL
>RpedHsp17.0-1
MLVINRLRIGLEVCAVKAPGFGEIRKEILKDMAVITGGEVIGDEVGIDLEKVTVKHLGQAGE
VIVNKGETFIIRGKGSEDEIKSRIAHIDLLISKAKNDFIREGLEERKGRLDSGVAIIYIGGYSEW
DVNERKDRVTDALNATKAAIDEGIVAGGGTA
>RpedHsp11.2-4
MGVEIVADALKMPCYTIAKNAGADASIVVSKVMDASGDIGYNALTNEYTNLIKAGIIDPTK
VVRTAINTAGRVASLLTSTEAVIADTGEKESNKEDSLDEEGRQARL
>RpedHsp59.5
MAGFPGSIAFDEYGRPFIIIKDQDKQKRLTGNDAIKSHILSAKAIARIVRTSLGPKGLDKLLVS
QDGDVMVSNDGATIMKQMDVDNEIAKLMVQLSQSQDDEIGDGTTGVVVLAGALLEQAD
VLLDKGIHPIRIADGFELAAKAALEHLDTISTKFPVDQNNLEPLIKTAMTTLGSKIVNKCHR
QMAEIAVKAVLSVADLEKRDVNFELIKVDGKVGGRLEDSILVKGVVIDKDFSHPQMPKVLK
DVKIAILTCPFEPPKPKTKHKLNVTSVEDYRALRKYEEEKFDQMVTQVKEVGTTLAICQWG
FDDEANHLLLKRELPAVRWVGGPEIELIAIATGGRIVPRFEELSPEKLGRAGIVREISFGTTKD
KMLVIEDCANSRSVTIFIRGSNQMIIDEAKRSMHDALCVVRNLICDDSIVYGGGAAELSCSIA
TAEKADTISTIEQYAFRAFSEALETIPLALAENSGLPPMQTVTSLRGRQIQEKNPALGVDCM
AKGTNDMKEQHVIETLKSKKQQIMLATQLVKMILKIDDVRTPNDIQL
>RpedHsp7.8-1
MGYDAKKDDYVNMMEQGIIDPTKVVRTALTDAAGVASLLTTAEAVLAEIPSKDKGPGGLP
PALGGAGGGGAFPQMADM
>RpedHsp7.7-2
MFKANTVLRQKNGYAKEVKFGNEAKRLLLSGVDKIANAVSVTLGPKVGRKILIRSPIDEFPI

TFIKVFNS

RpedHsp70:
>RpedHsp5.8-1
MPVSSNLPRGLLSFVIARSPSYTWMSTPGWLSEYIVKVCVCSVGIVVFLFIRA
>RpedHsp6.6-1
MVLGKMKETAEAYLGKKATHAVVTVPAYFNDAQRQATKDAGTISGLNVMRIINEPTAAAI
AYG
>RpedHsp18.4
MVNHFVQEFRRKYKKDLTQNKRALRRLRTACERAKRTLSSSTQASIEIDSLYEGTDFYTSITR
ARFEELNADLFRSTMEPVEKSLRDAKMDKAQIHDIVLVGGSTRIPKVQKLLQDFFNGKELN
KSINPDEAVAYGAAVQAAILHGDKSEEVQDLLLLDVTP
>RpedHsp29.9
MNILSSQTADAMADLTYLINTSNDHKIKINALLKRACIYMQSDDERASEDFSEAANLDPNN
PDVPHQRAQMFLLGERLENAVSEFHKAAELNPNSGLVQVQKLYAEYRYGTTQQDARMVA
KAKAGLHEAINKYPNTPEGYMLLAQVYNDSQEYDKAEATFDAGIKADPKNASILVHKGLL
YLQWNGEMDKAITMMEKGIAVDPKCEFAYETLASIEVQRGNLTRAVALFDKALPLAKSERE
ASHILSLRAAAQAQIVVAKTLGLDSILPSA
>RpedHsp74.9
MAAMSVIGIDFGNESCYVAVARAGGIETIANDYSLRATPSCVAFSGRNRILGVAAKNQMVT
NMKNTIYGFKRLLGRKINDPLIEEELQTLPFKVAPHLGNKIGIKVNYMEEEHTFTPEQITAM
LFTKLKEISEQALKTKVNDVVISVPSYFTNAERVALLDAAQITGLNVLRLMNETTATALSYGI
YKQDLPGPDEKPRNVVFVDCGHSTLQVSACAFNRGKLKMLASAYDHKLGGRDIDKILAD
HFSTEFETKYKIRPRTNPRAYLRLLTEVEKLKKQMSANSTTLPLNIECFMDDKDVHGDMKR
ADMEGLCAHIFHRVELCLKKCLEDSKLKLEEIHSVEIVGGSTRIPAIKQLIESVFKKTASTTLN
QDEAVARGCALQCAILSPAVRVRDFSIADVYPYPVCLSWDAHTKEDSVMEVFPQFHPMPFS
KVLTFYRRDNFDIKAYYNCDTPYPDRFIGQFTIKDVKPTPEGESSKIKVKVRINLNGIVGIVG
ASLVEKREAEGDEETMEVEQGNQNQSEQPQQNGQPDQQSLGGQLQSQFSENHFKRKGSK
FDSSNRCTDTSDYKFYNTASGYKRLKGNGGLHYKNGIYINEFVHDEFAPIDIEEAEENEKSD
KDKSDKKDGKKKKQITKTIDLPIDSLIHGYSQKELNDFQEFECKLIANDKQ
>RpedHsp5.4-2
MVVLRLMRAVMTPPAVSIPRERGATSRSRISLVDLLEVSPDRMAAWTAAP
>RpedHsp9.8
MSINPDEAVAYGAAVQAAILSGDQSSQIQDVLLVDVAPLSLGIETAGGVMTKIIERNTRIPCK
QTQTFTTYSDNQPAVTIQVYEGERVMTKD
>RpedHsp13.9-1
MAKAPAVGIDLGTTYSCVGVFQHGKVEIIANDQGNRTTPSYVAFTDTERLIGDAAKNQVA
MNPNNTIFDAKRLIGRRFDDVTVQSDMKHWPFEVISDGGKPKIQVQYKGENKTFFPEEISS
MVLTK
>RpedHsp8.6-1
MFVIICRNVNIPTKVTRTFTMSYSNDPRILIKVYEGERIMTKDDNLLGKFELAGIPKESKCFL
QIDVIFDIDANG
>RpedHsp35.4
MVLMKMRECAEAYIGKKVTNAVITVPAYFNDSQRQATKDAGTIAGLNVLRIINEPTAAAVA

YGLDKKGAGELNVLIFDLGGGTFDVSILTIADGVFEVRSTAGDTHLGGADFDNRMVRYFM
EEFKKKNRKDISDNKRALRRLSAACEKAKRTLSTATQASVEIDSLSDGIDLFSTISRAKFEELN
SDLFRGTLQPVEKAIRDSKISKDRIDEVVLVGGSTRIPKIQTLLMDFFGGKELNKSINPDEAVA
YGAAVQAAIIMGDSSDEVKDVLLLDVTPLSLGIETAGGVMTDLIPRNTTIPAKHSQIFSTYSD
NQPGVLIQVYEGER
>RpedHsp4.2-1
MIKDAEEFADDDKKRKEKVEARKELESYAYSLKNQ
>RpedHsp29.7
MAKTPAIGIDLGTTYSCVGVFQNGKFEIIANDEGKRRTRSYVAFTDRKCLIGDAAKHQTSV
NPNNTIFDAKRLIGRRFDDATVLSDMKRWPFEVINNGGKPKIQVKYKGETKSFYPEEISSMV
LRKMKETAESYLGKTVTNAVVTVPAYFNNSQRQATIDAGTISGLNILRIINEPTAAAISYGLD
KKVSDKRNVLIFHLGGGTFDVSILYIKHNKIKVKSTAGNTHLGGEDFDSRIIDHFVQEFKRK
YKKDISQNKSALWILRIA
>RpedHsp11.5-1
MAADNKLLGQFTLIGIPPAPRGVPQIEVTFDIDANGIVHVSARDKGTGKEQQIVIQSSGGLS
KDEIENMVRNAEQYAQQDKVKKDRVEAFNQAENIIHETESKMA
>RpedHsp8.9-2
MDLFRSTMKPVQKVLEDADMNKKDVDEIVLVGGSTRIPKVQQLVKEFFNGKEPSRGINPDE
AVAYGAAVQAGVLSGEQDTDS
>RpedHsp14.1
MNPQNTVFDAKRLIGRKYDDPKIQEDIKHWPFKVVNDCSKPKIQVEYKGEMKTFAPEEISS
MVLIKMKETAEAYLGSKVKDAVITVPAYFNDSQRQATKDAGAIAGLNVLRIINEPTAAALA
YGLDK
>RpedHsp7.4-4
MIREFKKYILELANALTQQILQFMLALHLLNNKYLCYFQVAYGAAVQAGVLSGEQDTDSIV
LLDVN
>RpedHsp30.8
MPAVGIDLGTTYSCVGVWQQGKVEIIANDQGNRTTPSYVAFTDTERLIGDAAKNQVAMNP
QNTVFDAKRLIGRKYDDPKIQEDLKHWPFKVVNDFSKPKIQVEYKGETKKFAPEEISSMVLT
KMKEIAEAYLGTKVKDAVITVPAYFNDSQRQATKDAGAIAGLNVLRIINEPTAAALAYGLD
KNLKGERNVLIFDLGGGTFDVSILTIDEGSLFEVKSTAGDTHLGGEDFDNRLVNHLAEEFKR
KYKKDLKTNARAVRRLRTAAERAKRTLSSSTEAT
>RpedHsp9.2-2
MNPKNTVFDAKRLIGRKYDDPKIQDDMKHWPFTVVSDGNKPKIQVEYKGETKRFAPEEISS
MVLTKMREIAEVYLGSKVN
>RpedHsp8.9-3
MTKDNHLLGKFDLTGIPPAPRGIPQIEVTFEIDANGILQVSAGDKGTGNREKIVITNDQNRL
TPEDIERMIKDAEKFADDD
>RpedHsp69.6
MVAVGIDLGTTYSCVGVWQHGKVEIIANDQGNRTTPSYVAFTETERLIGDAAKNQVAMNP
QNTVFDAKRLIGRKYDDPKIQEDLKHWPFKVVNDNLKPKIQVEYKGEMKRFTPEEISSMVL
TKMKETAEAYLGSKVRNAVVTVPAYFNDSQRQATKDAGAIAGLNVLRIINEPTAAALAYGL
DKNLRGEKNILIFDLGGGTFDVSVLTIDEGSLFEVKATAGDTHLGGEDFDSRLVNYLADEFK
RKYKKDIKSNPRSLRRLRTAAERAKRTLSSSTEASIEIDALYDGTDFYTKVSRARFEELCSDLF

RSTLQPVDKALQDAKLDKSSIHDVVLVGGSTRIPKIQSLLQHFFNGKALNMSINPDEAVAYG
AAVQAAILSGDSSSQIQDVLLVDVAPLSLGIETAGGVMSTIVERNTRIPCKQTQMFTTYSDN
QPAVTVQVFEGERVLTKDNNLLGTFDLSGIPPAPRGVPQIEVTFDIDANGILNVSAKEKSTG
KSRNIKIENNKGRLSKSEIDKMLRDAERYKAEDDHQRERISARHKFESYLFGVKQAAEEAG
RKLSDSDKQKIIRKCQEGIRWLENNTLADKEEYQDKLEQLQRECSPFMGKLHGRRGYGTQ
DDDSGPTIEEVE
>RpedHsp46.1
MPAVGIDLGTTYSCVGVWQNGKVEIIANDQGNRTTPSYVAFTESERLIGDAAKNQVAMNP
QNTVFDAKRLIGRRFDDPKIQEDIKHWPFKVINDSTKPKICVMYKNENKKFAPEEISSMVLT
KMREVAEAYLGRKVRDAVITVPAYFNDSQRQATKDAGAIAGLNVLRIINEPTAAALAYGLD
KNLRGERNVLIFDLGGGTFDVSILTIDEGSLFEVKSTAGDTHLGGEDFDNRLVNHLKDEFKR
KFRKDMSSNSRAVRRLRTAAERAKRTLSSSEASIEIDALFDGIDFYTKISRARFEEINADLFKS
TLKPVEKALNDAKLDKSSIHDVVLVGGSTRIPKIQTLLQNFFNGKSLNMSINPDEAVAYGAA
VQAGVLSGEQDTDSIVLLDVNPLTLGIETVGGVMTKLIPRNTVIPTK
>RpedHsp34.5
MDLFRSTLTPVERALNDAKLDKGSIHDVVLVGGSIRIPKIQKMLQDFFNGKTLNLSINPDEA
VAYGAAVQAAILSGDTSSAIQDVLLVDVTPLSLGIETAGGVMTKLIERNARIPCKQQQTFTTY
SDNQSAVTIQVFEGERAMTKDNNLLGTFNLNGIPPAPRGVPKIEVTFDLDANGILNVSAKD
SSTGKSEKITITNDKGRLSKSEIDKMLADAEKYKAEDDKQREKVQARNQLESYCFSLKQAA
EEAGSKLTDEDKKTVADRCAEVLSWLDSNSLAEKEEFEDKLKELQKVCGPIMTKLHQAGG
AKGPTVEEVD
>RpedHsp88.8
MMLKKAQEMAVISAGQAITEAVITVPGYFNQAERKAMLQAADLAGINVLQLINDYTAVAL
NYGIFRRKDFNESAQYIMLYDMGASSTTAAVISYQVVKTKEKGFVENHPQLSILGVGYDRTL
GGLEMQLRLRDYLAKKFNDLKKTPNDVFKNPRAMAKLFKEAGRLKTVLSANADHFAQV
EGLIDEVDFRLQVTRDEFEKLCSDLFERAKTPVLNALKTSGLSLDLVSQVILVGAGTRVPAVQ
EKLVSLVGSELGKNLNTDEAAVLGAVYKAADLSAGFKVKKFITKDGVIFPIQVTFERETEAG
PKLVKRTLFGSMNPYPQKKVLTFNKYLTDFEFNVGYGELDLEEKEIQNLGSLHLSKVSLSGV
AKAIAKHQSPKAEIKGIKAHFFMDESGLLKLSNVELVVEKTISEGDLNAAEESPLSKLGNTIS
KLFTSNSEEAPKEEKAKEEEKPVQEEKEEAKDSEKKEEKPADSNKTEESGEKDDKKPKETEP
PKPKAVLLKENITSKEESYILSDLNPAQLEEARSKLAKLNAIDEERRKRESTLNALESFVFETQ
NRLDTDEYVSAISPEERDNMKAAVSKVSDWLDEESMGSTAEVLSEKLKNLQKLTEAWFAR
VAEHRERPEALSALNTMLNGSRNFLVNIKNLTIQQAETPQGSVFTTVELETLEKTIKDTGDW
MDKVVDEQKKIPLWDNPKLTVKMISDKMASLDREVQYLVTKAKIWKPKPVEEKPKEVPVN
NGTENNATEEVVVENGEKSKAQDKSESVTIDKDSEEPNATKPEDQSEKVSDEEKHSEL
>RpedHsp8.2-2
MALLPLLSEILDDYRQPRLADLYDQHFGLGLLDNCTMMPTTILAAPLRSGYLRPWRNLLA
DQSGVSTIQSDKN
>RpedHsp4.2-3
MSILPYLLNELFDDYRHRPRPRDLLDQNFGLGLFN
>RpedHsp30.3
MSINPDEAVAYGAAVQAAILSGDQSSEIQDVLLVDVAPLSLGIETAGGVMAKIIDRNSRIPCK
QTQTFTTYSDNQPAVTIQVFEGERSMTKDNNLLGTFDLCGIPPAPRGVPKIEVTFDMDANGI
LNVSAKEISTGKSKNIVIQNDKGRLSKEEIDRMVNDAERYKAEDEKQKLRVTARNKLESYV

YGVKQAVEECGNKLSEPEKMKANSVCDKTIKWLEQNQMAEKEEYEYKFQEVQKECSPLM
TKLHGGSSYGYGQERGENYHSGPTVEEVD
>RpedHsp14.0-3
MSNIFGLYVGNSTASIAIYKEEGKVEVLANQAGERVTPATVSISHQEKVVGLTTVSFSRAAST
VTNNKRLLDTDINLDDLEQYVAQSSVPLITENSLAYNISEPDRKPYVYTPAQVAVLIFQKLYN
IGV
>RpedHsp13.9-2
MIKVLTDHLVEEFSRKYYLDPNESRKSLWKFRNAAQIILHTLSTLPTSSYFIESACEGVDFNVT
VSVARLNSLLSPLLPTFADPIHKALQQAQLSSSQISKVILCGGCLKVPKIRAYVSSFFEC
>RpedHsp81.8
MKDSPIELAHRHTGELIVSNDEEQPTGGETEPSNRSISPSSSYSSQPDCLVQAFNSQLCVLSPR
MSPVGCSSECSVELGKETDNLIEEIYIAKHSIAKGIIYQNNIEAYAAKIPPNVPVVELQDGKM
QNNNIDNEKKNNFVEKVIVNVEQERKANRSEDFDVVVAIDLGTTYSGYAFAYTKNPSDKQI
HMMRQTEGSDRGLNNQKVPTVLLLTPEEKFHSFGYAARDFYHDLDPSDAKQWLYFDKFK
MHLHNNMDVNRESQVAAQNGQTISALTVFAHTLRYMKWQVEKELRDQGRVPSVRWVIT
VPALWTQQAKQFVREAAYMADISSPDTSDHLLIALEPEAASVCCRNLHFDQIIQLNSNRLRS
GSYMVVDCGGGTVDITVHTVSAQTGTLRELHKATGGPCGSIGVDKEFNDLLDTVFGTSFM
RRFQQERPAAYNELMLSFEARKRSTTSFRSTPLNIFPPFAFIDFFRKVSGIEVEAAVKEYGRPEL
TWSNEGILKIHPSLMYQLFQPTLNKIIEHVEAVLSSRRIDDSISHLFLVGGFSESQILQEAIRDN
FSNRVHIIIPQAVSLSVLKGAVLYGLNPTIVSSRRVTHTYGLGVVKPFISGFHPLDKLVIRGGQ
KWCVDVLERLVESGQAVSVGQIVSKKFTPASMNQREIVLQIYATLSKTAQFITDNNVYKCGL
LRLALPEASSEHKLREIVVSLVFGGTEVTAFAVDSSTGYSVKTTLEFMD
>RpedHsp18.1
MTKDNNLLGKFELTGIPPAPRGVPQIEVTFAIDANGILNVTAIEKSTGKENKITITNDKGRLS
KEEIERMVNDAEKYKAEDDKQKAVIQAKNTLESYCFNMKSTVEDEKLKDKIPEADKNTILE
KCNEVIRWLDANQLAEKEEFEHKQKELEQLCNPII
>RpedHsp14.4-1
MTKDNNLLGTFDLTGIPPAPRGVPKVEVTFDLDANGILNVSAKENSTGKAKNIRIENNKGR
LSKEEIERMVNEAERYKAEDDAQRERIAARNQLEGYIFSIKQAIDEAGDKWSEEDKNTARA
KCDEAMR
>RpedHsp20.8
MSINPDEAVAYGAAVQAAILSGDQSSQIQDVLLVDVAPLSLGIETAGGVMTKIIERNSRIPCK
QTQTFTTYSDNQPAVTIQVFEGERAMTKDNNLLGTFDLTGIPPAPRGVPKVEVTFDLDANG
ILNVSAKENSTGKSKNIRIENNKCRLSKEEIERMVNEAERYKAEDDAQRERIAARNQLEGYI
FSIK
>RpedHsp11.1-2
MTLLNKFRMIWVCPIVLIALIHCVEKTTAISVMSIDFGTEWMKVAIVSPGVPMEIALNKESK
RKTPVAVAFRDGERTFGEDVLTIGVRFPKQCYVYLLD
>RpedHsp6.3
MALASLLNELLLDELRRPTLADLYDQHFGLGLHNHEALHPASTYLLSPLRSGYLRP
>RpedHsp4.6-4
MLDDYRRNPFANLYDQNFGLGLLNDATLHPAHSLPASPLRCG
>RpedHsp4.9-1
MSLLPFVSAMLDDYRRNPFVDLYDQNFGLGLLNDAALHPAHSLL

RpedHsp90:

>RpedHsp4.6-1

MKENQKHIYFITGENKDQVANSSFVERVKKRGFEVVYMT

>RpedHsp5.4-1

MKDILEKKVEKVVVGNRLVDSPYCIVTSQYGWTANMERILKAQALRD

>RpedHsp16.9-1

MKDILDKKVEKVVVSNRLVDSPCCIVTSQYGWTANMERIMKAQALRDSSTMGYMAAKKH
LEINPDHAIVETLRQKAEANKNDKAVKDLVLLLFETSLLSSGFGLEDPQVHASRIYRMIKLG
LGIDEEEAPGQEETPDVEMPPLEASAEDASR

>RpedHsp83.6

MPEDVEAADVETFAFQAEIAQLMSLIINTFYSNKEIFLRELISNSSDALDKIRYESLTDPTKLE
NGKELYIKIIPNKNDRTLTIIDTGVGMTKTDLVNNLGTIAKSGTKAFMEALQAGADISMIGQ
FGVGFYSAYLVADRVTVCTKHNDDEQYIWESSAGGSFTVRPDPGEPLGRGTKIILSVKEDQE
EFLEERKIKEVVKKHSQFIGYPIKLLVEKERDKEISDDEGEECNDDSEKAEEGEDKEKGDQPK
VEDVGEDEEGDDANKEKSKKKKKKIKEKYTEDEELNKTKPIWTRNPDDITGDEYGEFYKSL
TNDWEDHLSVKHFSVEGQLEFRALLFVPKRAPFDLFENRKKRNNIKLYVRRVFIMDNCEDII
PEYLNFIKGVVDSEDLPLNISREMLQQNKILKVIRKNLVKKCIELFEELTDDKEAYKKFYEQF
SKNIKLGIHEDSTNRKKLAELLRFHTSASGEDACSLKDYVGRMKDNQKHIYFITGENRDQV
AHSAFVERVKRRGFEVIYMTEPIDEYVVQQMKEYDGKQLVSVTKEGLELPEDEEEKKKREE
DKAKFENLCKIMNDILEKKVEKVIISNRLVDSPCCIVTSQYGWTANMERIMKAQALRDTST
MGYMAAKKHLEINPDHPIIEVLRQKAEVDKNDKAVKDLVILLFETSLLSSGFSLEDPQVHAS
RIYRMIKLGLGIDEDDSVAIEEEEQAEELPSLEGSTEDASRMEEVD

>RpedHsp37.3

MKKYIILGLGLLLLLLGAARAEDDVLEETVNDDLGASREGSRTDDQVVEREENAIKLDGLNV
AQIKELRDKAEKFAFQAEVNRMMKLIINSLYRNKEIFLRELISNASDALDKIRLLSLTEPGALS
TKEELSIKIKADKEGKLLHITDSGIGMTKADLVNNLGTIAKSGTAEFLNKLQDEGTSSQDTM
DLIGQFGVGFYSSFLVADRVIVTTKHNDDKQLIWESDAASFSISEDPRGDTLKRGTQISLFLK
DEATDYLEVGTLRNLVKKYSQFINFPIFLWADKTVTVEEPLEEGEEPEKKEETDEDVGVEDE
KEEDKPKTKSVQKTVWEWER

>RpedHsp40.0

MARWGEGDPRWIVEERPDATNVNNWHWTEKNACGWSKDKLKELLVGLKLESEVADVTL
TEMEKIDGEAVANNRKGKLIFFYEWDMSLIWHGRLKGGTGEHEGKIKIPNLSEENDVSEIEV
SVTLKESNEEGEIIKQYLHHQGSEKIRERLGLYISSLKEEFSKGMILPKKDEILNDNKEAQQIS
NLSKGFSNKMHMNQVNNKAQTTNNINKRKIDTTTLELSQTFQCRGEDFYNAMTKPEMVS
AFTNGPAVIDSKVGGRFELFGGNIIGEFTELVQDKKITQRWRLKGWPEGHYSTVSIVIDQQE
DHTLVKMSQSDIPKSDMEGTKENWSRYYWDAIKRTFGWGYFM
